# Family therapy and cognitive behavior therapy for eating disorders in children and adolescents in routine clinical care: a systematic review and meta-analysis

**DOI:** 10.1007/s00787-024-02544-1

**Published:** 2024-08-27

**Authors:** Gro Janne Wergeland, Ata Ghaderi, Krister Fjermestad, Pia Enebrink, Lillan Halsaa, Urdur Njardvik, Eili N. Riise, Gyri Vorren, Lars-Göran Öst

**Affiliations:** 1 Department of Child and Adolescent Psychiatry, Division of Psychiatry, Haukeland University Hospital, Bergen, N-5021 Norway; 2 Department of Clinical Medicine, Faculty of Medicine, University of Bergen, Bergen, Norway; 3 Division of Psychology, Department of Clinical Neuroscience, Karolinska Institutet, Stockholm, Sweden; 4 Department of Psychology, University of Oslo, Oslo, Norway; 5 Innlandet Hospital Trust Norway, Ringsaker, Norway; 6 Department of Psychology, University of Iceland, Reykjavik, Iceland; 7 Department of Child and Adolescent Psychiatry, District General Hospital of Førde, Førde, Norway; 8 Department of Psychology, Stockholm University, Stockholm, Sweden

**Keywords:** Eating disorders, Children and adolescents, Effectiveness, CBT, Family therapy, Meta-analysis

## Abstract

Family therapy for eating disorders (ED) is well-established and represents the treatment choice for ED in children and adolescents according to guidelines, with cognitive behavior therapy (CBT) as a second line treatment. There is limited knowledge about how these treatments work in routine clinical care. The goal of the present meta-analysis is to investigate the effectiveness of family therapy and CBT for various EDs in children and adolescents when carried out in routine clinical care. Ovid MEDLINE, Embase OVID, and PsycINFO were searched for articles published until December 2023. The outcome of family therapy and CBT, methodological quality, risk of bias, and moderators of treatment outcome were examined and benchmarked by meta-analytically comparing with ED efficacy studies. Forty-four effectiveness studies comprising 3251 family therapy or CBT patients were included. Large to very large within-group effect sizes (ES) were found for ED-psychopathology (0.80) and weight measures for AN (1.64) at post treatment. The attrition rate was 15%. Risk of bias was considerable. Moderate to large ES were found for family therapy and CBT, respectively. The benchmarking analysis showed that effectiveness studies had comparable ESs to efficacy studies (0.80 and 0.84 for the ED-psychopathology at post treatment). The findings support family therapy and CBT for ED in children and adolescents as effective treatments when delivered in routine clinical care, with effects comparable with those found in efficacy studies. The evidence needs to be interpreted with caution because of the risk of bias in a high proportion of studies.

PROSPERO [CRD42023441794].

**Supplementary Information:**

The online version contains supplementary material available at 10.1007/s00787-024-02544-1.

## Introduction

Anorexia Nervosa (AN), Bulimia Nervosa (BN), Binge-Eating Disorder (BED), and Other Specified Feeding and Eating Disorder (OSFED) represent the main eating disorders (ED) defined in the Diagnostic and Statistical Manual of Mental Disorders, 5th Edition (DSM-5) [1]. They are characterized by persistent disturbances of eating behaviors and a core psychopathology centered on food, eating, and excessive concerns about weight and shape. The age of onset for ED is most frequently during mid to late adolescence [2, 3]. The prevalences of ED in children and adolescents aged 11–19 years have been reported to be 1.2% (boys) and 5.7% (girls) [4, 5], with an increasing incidence during the Covid-19 pandemic [6]. ED can be life-threatening illnesses and are associated with significant impairments in psychiatric and somatic health, quality of life, and delays in development [7, 8]. Among adolescents with an ED there is common co-occurrence with other psychiatric disorders, particularly depression (up to 50%) and anxiety disorders (up to 35%) [9, 10]. Due to the serious nature of ED and their somatic and mental health consequences, early identification and treatment is important.

Clinical practice guidelines and consensus statements have been developed for the management of ED in children and adolescents up to 18 years [11–14]. Both commonalities and differences are found in the recommendations on psychological therapies for youth with ED among the clinical practice guidelines internationally [15]. For children and adolescents with AN and BN eating disorder-focused family therapy is the psychological treatment with the strongest evidence-base [10], and is considered the first choice of treatment across the guidelines, whereas cognitive behavior therapy (CBT) for eating disorders could be considered if family therapy for ED is not feasible or accessible, has been ineffective, or is undesired due to patient or family preferences [e.g., 12, 13]. For children and adolescents with BED treatment with CBT for eating disorder is recommended, and for youth with OSFED it is recommended to offer the ED-treatment that the OSFED most closely resembles [12].

Involvement of the family in the treatment of ED in children and adolescents is based on models and techniques from several schools of family therapy, as well as systemic and behavioral therapeutic approaches. The two main models of family therapy for ED in children and adolescents are the Maudsley family therapy [16], and family based treatment (FBT) [17]. While some differences exist both models are phased, and a major aspect of both approaches is empowering parents to mobilize the family system and family resources they possessed prior to the onset of the disorder, re-implement them in the family system, and to encourage behavioral change in their child [16, 18]. The treatment can be implemented in routine clinical care [19], be adapted for higher levels of care [20], and can be delivered in different formats, including multi-family therapy [21]. Following family therapy for ED the remission rates among children and adolescents with ED are reported to be 40–50% [10, 22]. These numbers indicate that for a significant proportion of children and adolescents with eating disorders high levels of eating pathology persist at the end of treatment. Whereas Maudsley family therapy and FBT are well-established treatments [10] and the leading psychological therapies for children and adolescents with ED, they may not be effective or suitable for all. A second-line therapy that could then be considered is cognitive behavior therapy for eating disorders (CBT-ED) [12].

CBT-ED is an individual therapy that addresses the core psychopathology of eating as well as weight and body shape concerns through behavioral reduction of restraint, establishing regular eating and normalization of weight among underweight patients, and cognitive interventions to address dysfunctional beliefs and practices [23, 24]. Enhanced CBT (CBT-E) is a manual-based and trans-diagnostic version of CBT, where an individualized formulation of the patient’s difficulties guides the treatment [25, 26]. CBT for the eating disorders is widely recognized as the treatment of choice for ED in adults, with good recovery rates particularly for non-underweight individuals [24]. CBT-E has been adapted for use with children form the age of 11 and may include the involvement of parents given the child’s age and circumstances [27, 28], and for use with those who required more intensive care such as daycare or inpatient treatment [29]. CBT has been found effective for children and adolescents with a remission rate of up to 50% [22].

Data on the effectiveness of family therapy and CBT for ED when delivered in routine clinical care are emerging, however, the data are scarce compared to the empirical support from efficacy studies [10]. Examining the effectiveness is important as evidence-based therapies may perform differently in routine clinical care compared to delivery in the research settings [30]. Studies conducted for the purpose of establishing efficacy are designed to have high internal validity, e.g., by using rigorous inclusion and exclusion criteria, randomizing participants to conditions, and having highly trained therapists. This methodological rigor of efficacy trials, aimed at maximizing experimental control, may reduce external validity. There have been questions raised about the transferability of results to routine clinical care where patients, therapists, and treatment context may differ from those in efficacy studies [31, 32]. Therefore, studies in less controlled routine clinical care settings, at sites beyond those where the initial evidence was derived, have been called for [33]. Also, the routine clinical care setting is a crucial service site since the majority of children and adolescents with ED will seek and receive their treatment there [34]. Thus, it is important for clinicians to know what outcome to expect from the recommended treatments of ED when delivered in routine clinical care, and how results fare in comparison with outcome in specialized university research settings.

Previous meta-analyses focusing on the effectiveness of evidence-based treatments for children and adolescents when delivered in routine clinical care have reported treatment outcomes comparable to outcomes from efficacy studies conducted in university research settings. These meta-analyses have examined effectiveness studies for children and adolescents with internalizing disorders [32], externalizing disorders [35], and for children with autism spectrum disorders [36]. To the best of our knowledge, no meta-analysis of effectiveness studies of family therapy and CBT for ED in children and adolescents has been published. In the most recent evidence-based update on psychosocial treatments for ED in children and adolescents results across 31 studies were examined for various interventions [10]. Thus, a meta-analysis on current state of the effectiveness of family therapy and CBT for children and adolescents with ED in routine clinical care is warranted.

Previous meta-analyses of psychological treatments have found different moderators of the effect size (ES). In the present meta-analysis we will use five categorical variables. The first is design. A meta-analysis by Hilbert et al. [37] reported that study design was not a significant moderator of the primary outcome. Since our meta-analysis uses pre-post ES and includes both RCTs and Non-RCTs it is important to investigate this variable. The second is statistical analysis. Some meta-analyses have found no difference in ES between intent-to-treat (ITT) and completer analysis [e.g., 38, 39], and others that completer analysis yielded a higher ES [e.g., 40]. Thus, from a methodological point of view, this is an important moderator to assess. The third is risk of bias. A meta-analysis on AN [38] described that studies with low RoB yielded higher effect size than studies with high RoB, whereas other meta-analyses have reported that RoB was not a significant moderator [39, 40]. Thus, RoB is included as a moderator. The fourth is treatment format. Meta-analyses by Davey et al. [41], Hilbert et al. [39], and Linardon and Wade [42] reported treatment format to be a significant moderator of outcome. The fifth is continent. Previous meta-analyses investigating this variable have reported different results. For example, Cuijpers et al. [43] found that studies from North America yielded higher ES than studies from Europe, whereas Öst et al. [44] and Wergeland et al. [32] reported that studies from Europe yielded higher ES than studies from other continents.

There are also some continuous variables of interest as potential moderators. We considered five. The first is mean age. Hilbert et al. [39] found that lower age of the sample was associated with better outcome, Linardon et al. [45] and Murray et al. [40] that age was unrelated to outcome, whereas Svaldi et al. [46] and van den Berg et al. [38] reported larger effect size for samples with older patients. The second is the percent of females. Hilbert et al. [39] found that higher proportion of females in the study was associated with better outcome, whereas Linardon [47] reported that sex was not related to the outcome. The third is pre-treatment severity. Hilbert et al. [39] found that the lower the BMI and the higher the number of binge-eating episodes at pre-treatment the better was the outcome, whereas Öst et al. [48] did not find that pre-treatment severity of ED psychopathology was a significant moderator. The fourth is the methodological quality of the included studies. Quality has in previous meta-analyses been found to be associated with lower ES [49] as well as with higher ES [50]. The fifth is the amount of therapy, measured as months of therapy and hours of treatment. Amount has in some meta-analyses been found to be a positive moderator [36, 51], but Svaldi et al. [46] did not find that the duration of treatment was related to the outcome.

The present study aimed to add information to the literature by providing a meta-analysis on the effectiveness of ED-focused family therapy and CBT for ED children and adolescents, i.e., treatments that are recommended according to international guidelines for ED, when these are carried out in routine clinical care. Studies in which patients are referred for treatments through usual clinical routes, treatments are delivered by practicing clinicians, and as part of routine clinical care were included. Both non-randomized and randomized trials were included to ensure comprehensive coverage and because both designs are commonly used in effectiveness studies [31]. The specific aims were: First, to examine the effectiveness of ED focused family therapy and CBT for ED in children and adolescents. Second, to evaluate methodological stringency and risk of bias in the effectiveness studies and investigate potential moderators of treatment outcome. Third, to compare the effectiveness of family therapy and CBT for ED, and fourth to compare the outcome of these treatments delivered in routine clinical care with that reported in efficacy studies for ED.

## Methods

The meta-analysis was pre-registered at PROSPERO [CRD42023441794], and was conducted following the Preferred Reporting Items for Systematic Reviews and Meta-Analyses guidelines (PRISMA) [52]. For details see Supplementary information (SI) 1. The meta-analysis was designed according to the PICOS acronym in the following way:

- *Population*: children and adolescents with an ED diagnosis.
- *Intervention*: any format or variations of family treatment (FBT, FT-AN, multi-family treatment), or any format (Individual, Group, Self-help, Guided self-help) of CBT (CBT-ED, CBT-BN, or Transdiagnostic CBT).
- *Comparison*: within-group change, i.e., pre vs. post/follow-up data.
- *Outcome*: primary (ED-psychopathology symptoms, weight) and secondary (depression, remission).
- *Study design*: RCTs and pre-post/Non-randomized studies of intervention (NRSI).

### Literature search

Studies were identified by systematic and comprehensive literature searches of the electronic databases Ovid MEDLINE, Embase OVID, and PsycINFO from the start of the data bases to April 14, 2023, with an updated search on December 8, 2023. The list of search terms was generated by the authors in collaboration with a university librarian. Both subject headings and free text words for the following search terms to search the databases were used: Family therapy and variations thereof; Cognitive behavior therapy and variations thereof; ED (including the different eating disorders); the design of the study; age group (up to 18 years). For details on the electronic search see SI 2.

The abstracts were read by four pairs of authors independently of each other to decide whether a study warranted a more detailed reading. Full-text articles were retrieved if there was any indication of a target group of patients receiving the family therapy for ED or CBT in a routine clinical care setting. Additionally, a manual search was performed by reviewing the reference lists of potentially eligible articles. In total, 339 full-text articles were considered for inclusion. The final decision for article inclusion was made using a stricter set of inclusion and exclusion criteria detailed below. Disagreements were resolved by consensus discussion among the authors and/or consultation with the last author. In cases where there was insufficient information provided for the inclusion criteria to be applied or there were insufficient details reported on the outcomes, the corresponding author was contacted to request inclusion in the meta-analysis.

#### Inclusion criteria

To be included a study had to:

1. Be published, or in press, in an English language journal.
2. Have participants diagnosed with an eating disorder according to DSM (III and later) or ICD (10 or 11) with focus on AN, BN, BED, Other specified ED (atypical AN, BN and BED, and purging disorder) and avoidant restrictive food intake disorder (ARFID). Samples selected based on a transdiagnostic perspective were also included.
3. Be testing any format of family therapy or CBT.
4. Have participants referred for treatment through usual clinical routes.
5. Be an effectiveness study, i.e., carried out in a routine clinical care setting such as a community mental health center, at patients’ homes, etc.
6. Have therapists who are practicing clinicians for whom provision of service is a substantial part of their job.
7. Have a treated sample consisting of at least 10 participants.
8. Have a maximum mean sample age of 18 years, and a maximum participant age of 20.
9. Provide a continuous or dichotomous measure of the principal disorder treated, with data making it possible to calculate effect size.

#### Exclusion criteria

1. The study is a secondary analysis of a previously published study. Separate follow-up studies to the basic study are included to provide follow-up data.
2. The study is an evaluation of a service where the results for individual disorders cannot be extracted.
3. The study is testing a combination of family therapy for ED/CBT and another, e.g., pharmacological treatment and all participants in that condition receive both treatments. Also, studies selecting a sample of patients fulfilling criteria for an eating disorder and another psychiatric disorder, e.g., depression, are excluded.

### Categorization of studies

To be categorized as an effectiveness study, participants had to be referred through ordinary clinical channels (or self-referred), the treatment was carried out in routine clinical care settings (or in patients’ homes for internet-based treatment), and the therapists were ordinary clinicians who work with a caseload of patients with different diagnoses.

Studies with children and adolescents diagnosed with AN, BN, BED, EDNOS or OSFED were included. In addition, we included a number of studies that had a mix of the above eating disorders and presented combined results. For the comparison between treatment methods we combined CBT, CBT + App, and CBT-Enhanced (CBT-E) to the category *CBT*, and FBT, FT-AN/BN, and Multi-family therapy (MFT) to the category *family therapy for ED.*

### Potential categorical moderators

An a priori requirement for including any potential categorical or continuous moderator in the analysis was that at least 70% of the studies provided information on that variable, as lower rates would probably lead to questionable representativity. Design was categorized as either RCT or NRSI/Pre-post studies. Statistical analysis was categorized as completers (if dropouts were deleted) or as intent-to-treat (ITT, if all randomized or starting participants were included in the statistical analysis). Risk of bias (RoB) was based on a summary evaluation of the domains rated for the different designs (see below) and the studies were categorized as low, moderate, or high RoB. Treatment format could either be individual, family, or a combination of individual and family. The location in which the study was carried out was categorized as Africa, Asia, Australia, Europe, North America, or South America.

### Potential continuous moderators

The following variables were used as potential continuous moderators: mean age, percent females, pre-treatment severity (calculated as a percentage by dividing the sample mean with the maximum score possible of the rating scale applied), methodology score (see below), and amount of therapy. The data were extracted using a pre-designed coding scheme and a scoring manual including the variables of interest. The data were extracted and categorized independently by the pairs of authors and any disagreements were solved after consensus discussion.

### Methodological quality

#### The psychotherapy outcome study methodology rating scale (POMRS)

The POMRS consists of 22 items covering various important aspects of the methodology in psychotherapy outcome research [53]. Each item is rated on a 3-point scale (0 = poor, 1 = fair, and 2 = good), and each step has a written description. The total score can vary from 0 to 44 points. Since all items do not apply to all studies, the total score was recalculated as a percentage of the maximum score possible for the individual study. The internal consistency of the scale was good with a McDonald’s ω of 0.80. The inter-rater reliability of the scale (between GJW and LGÖ), based on 20% randomly selected and blindly rated studies, was ICC = 0.98 (95% CI 0.94–0.99, *p* = 0.0001), which according to Cicchetti [54] is excellent.

#### Risk of bias

The Cochrane Collaboration tool for assessing RoB [55]was used for RCTs and the RoB in NRSI (ROBINS-I) [56] was used for NRSI and pre-post studies. An overall classification of the studies was done for RCTs into the categories high, moderate (some concerns), or low RoB. For the NRSI and pre-post studies the categories low, moderate, and high (serious or critical) RoB were used. The rating of the studies was done by two authors (GJW and LGÖ) and differences were discussed to reach consensus.

### Effect size measures

#### Primary outcome measures

The first primary measure was scores on a validated semi-structured interview, or rating scale of eating disorder psychopathology. The Eating Disorders Examination (EDE) [57] is an interview-based assessor rating and different versions were used in 13 studies. The Eating Disorders Examination-Questionnaire (EDE-Q) [58] was applied in nine studies. The Eating Disorder Inventory-2 (EDI-2) [59] was used in seven studies, and EDI-3 [60] in four studies.

In studies on AN, weight is a commonly used primary outcome measure. This was assessed as per cent expected body weight (% EBW), defined as percentage of the expected weight corresponding to the 50th percentile for gender, age and height according to the Center for Disease Control growth charts in 21 studies, Body Mass Index (BMI) defined as kg/m^2^ was used in 14 studies, and % median BMI was calculated using charts from the World Health Organization (WHO) for age, height, and gender, and used in eight studies. In addition, we planned to assess binge eating episodes and compensatory behaviors but since very few studies provided this information it is questionable if the information extracted would be representative for the entire body of studies.

#### Secondary outcome measure

Depressive symptoms were considered in 15 studies, and ten studies used the Children’s Depression Inventory [61], three studies used the Beck Depression Inventory (BDI or BDI-II) [62, 63], and two the Mood and Feelings Questionnaire [64].

Another planned secondary measure was remission. However, only 15 studies (28%) provided such data, and this was not considered representative for all included studies.

### Meta-analysis

To obtain as many effectiveness studies as possible, both RCTs and pre-post/ NRSI trials were included in the meta-analysis since within-group ES can be calculated from both types of studies. ES were calculated as (Mpre – Mpost)/SDpre according to a recommendation by Lakens [65], since there is good reason to assume that the interventions influence not only the means but also the standard deviations. The mean ES was computed by weighting each ES by the inverse of its variance. ITT data were used when a study provided those, otherwise completer data were used.

Before pooling, the effect sizes were screened for statistical outliers, defined as being outside M ± 2SD. On the ED-psychopathology measures there was one outlier at the post-treatment assessment and one at follow-up. On the weight measures there was one outlier at follow-up. For these ESs, winsorizing [66] was used by reducing outliers to the exact value of M + 2SD. The Comprehensive Meta-Analysis v.4 (CMA) [67] software was used for the analyses and Hedges’ *g* was calculated to correct for small sample sizes. A random effects model was used since it cannot be assumed that the ESs come from the same population. Lipsey [68] described an empirically developed rule-of-thumb for considering an ES as small (≤ 0.32), moderate (0.33–0.55), and large (0.56–1.20). Also, Sawilowsky [69] denoted ESs as very large (1.20–1.99) and huge (≥ 2.00).

Sensitivity analysis was done for the primary outcome measure ED-psychopathology in three ways to test the robustness of the pooled ES. First the pre-post correlation was varied from 0.1 to 0.9 and then the effect of the different ED-psychopathology measures was tested by deleting each of them not being EDE or EDE-Q. Third, the pooled ES was calculated by removing one study at a time.

Proportions were analyzed in CMA. The values of the individual studies were transformed using logit transformation and the statistical analysis was done on the transformed proportions using the random effects model. Then the pooled proportion and its 95% confidence interval was back-transformed to a proportion.

Heterogeneity among ESs was assessed with the Q-statistic and the prediction interval. The true effect size in 95% of all comparable populations will fall within this interval [70]. Publication bias was assessed with funnel plots, Egger’s regression intercept [71] and the trim and fill method described by Duval and Tweedie [72]. Moderator analyses of categorical variables were done with subgroup analysis using the mixed effect model and of continuous variables with meta-regression using the random effects model.

### Efficacy studies for comparison

The recent comprehensive evidence-based update by Datta et al., [10] were consulted to obtain the efficacy studies to be used in a comparison with effectiveness studies. From this the RCTs of family therapy for ED and CBT recommended by the treatment guidelines reviewed in the introduction were listed. Since this update included both efficacy and effectiveness studies, those RCTs we had already included in the body of effectiveness studies were deleted. This resulted in 15 RCTs for our comparison and the references are listed in the SI 3. This type of benchmarking in which ES for effectiveness and efficacy studies are statistically compared using a meta-analysis software has previously been done in three similar meta-analyses on effectiveness studies in children and adolescents [32, 35, 36] and five in adults [44, 48, 73–75].

As for the effectiveness studies, data were extracted for the type of primary outcome measure most frequently used in both types of studies (some ED-psychopathology measure and weight in AN), at post-treatment and follow-up assessment separately. To compare the two categories of studies on background and treatment variables, data were also extracted on mean age, proportion of females, pre-treatment severity, comorbidity (% of the sample having at least one comorbid disorder), medication (% of the sample that at pre-treatment was prescribed a psychotropic drug), treatment time (in 60 min units), and attrition rate (% dropout of patients who participated in at least one session). Other variables were not reported systematically (or not at all) in a large enough proportion of studies, which precluded inclusion as a background variable. Since the result tables will entail many statistical tests, the Holm-Bonferroni correction was used to control the family-wise error rate [76].

### Power analysis

The number of studies and treatment conditions, which is the unit of analysis in the overall comparison of effectiveness and efficacy studies, were as follows: effectiveness studies 44/53 and efficacy studies 15/20. This yields a total number of 59 studies and 73 treatment conditions with an average of 54 participants per condition. According to the formulas for power analysis in meta-analyses by Valentine et al., [77], with these figures we would have a 99% power to detect an ES of 0.20, assuming a high heterogeneity.

## Results

### Description of the effectiveness studies

**Fig. 1:**
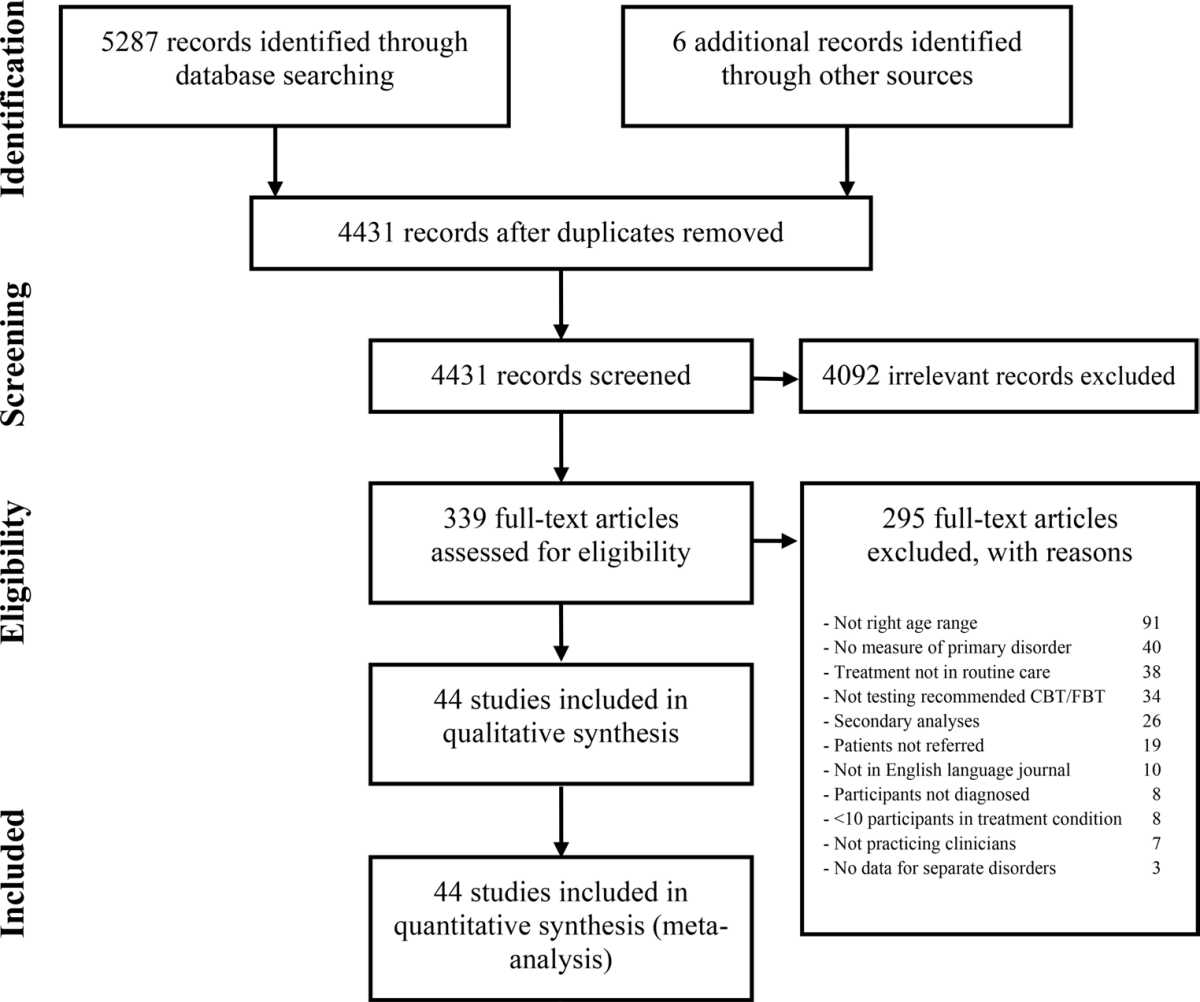
Flow-chart of the inclusion of studies

A total of 44 studies comprising 53 treatment conditions were included. A flow-chart of the study inclusion is shown in Fig. 1. References to the included studies are provided in SI 4.

#### Background data

Background data for the included studies are displayed in Table 1. The conditions came from the following continents: Europe 24, North America 16, Australia 12, and Asia 1. The number of conditions for the different ED were: AN 32, BN 1, EDNOS 1, and Mixed 19. The total number of participants receiving treatment in the studies was 3251 (range 10–290), with 94.1% on average being females. The mean age across the studies was 15.4 years (SD 0.5; range 14.1–17.9). The prevalence of comorbid psychiatric disorders was only reported in 58% of the conditions with a mean of reported disorders being 42.9% (SD 7.7) in these studies, and use of psychotropic medication in only 42% of the conditions with a mean of 27.4% (SD 39.2). The pre-treatment severity on an ED-psychopathology measure could be calculated for 77% of the conditions and the mean was 49.3% (SD 29.1).

**Table 1:** Background data for the included studies

Study	Eating disorder	Country	Treatment	*N*	% Declining tx	Dur. months	Previous Tx %	% Severity	% Female	Mean age	% medicated	% comorbidity
Accurso, 2018	AN	USA	FBT	11	21.4	6.4	27.0	33.3	100.0	15.5	18.0	45.0
Anastasiadou, 2020*	AN, BN, BED, O	Spain	CBT + App	53	24.3	39.6		46.7	90.6	17.3	66.0	41.5
Anderson, 2017	AN	USA	FBT Tele	10	52.4			40.0	80.0	16.1	30.0	50.0
Bentz, 2021	AN	Denmark	FBT	157	6.0			46.7	91.7	14.3		26.8
Calugi, 2019	AN	Italy	CBT-E	62		23.0		66.7	96.8	16.4	0.0	54.0
Chew, 2021	AN	Singapore	FBT	65	15.6	6.6			95.3	14.4	9.8	13.9
Coelho, 2019	AN	Canada	FBT	62					90.0	14.6		22.6
Couturier, 2010	AN	Canada	FBT	14		8.0	42.9	40.0	100.0	14.0	36.0	7.1
Craig, 2019	AN, BN	UK	CBT-E	54	33.3	24.7	59.3	56.7	96.3	15.5		
Dalle Grave, 2014	AN	Italy	CBT-E	27	15.6	24.0	100.0	61.7	96.3	16.0		
Dalle Grave, 2015	BN, BED, EDNOS	Italy	CBT-E	68		20.4		60.0	97.1	16.5		
Dalle Grave, 2019	AN	Italy	CBT-E	49	3.9	11.4		46.7	100.0	15.5		
Dalle Grave, 2020	AN	Italy	CBT-E	74	26.0	22.8	100.0	66.7	98.6	16.5		
Dalle Grave, 2023	AN	Italy	CBT-E	61	29.4	12.0		48.3	98.4	15.8		
Eisler, 2016*	AN, EDNOS	UK	FBT	82	33.2	7.0	12.9	46.7	93.9	15.7		12.2
Eisler, 2016*	AN, EDNOS	UK	MFT	85	33.2	7.0	19.5	41.7	88.2	15.7		2.4
Gabel, 2014	AN	Canada	MFT	25			92.0	51.7	100.0	14.1	72.0	48.0
Geist, 2000*	AN, EDNOS	Canada	FBT	12	0.6		0.0	39.6	100.0	14.3		
Geist, 2000*	AN, EDNOS	Canada	FGP	13	0.6		0.0	48.9	100.0	14.9		
Gelin, 2015	AN, BN	Belgium	MFT	82			28.0	44.6	97.6	16.0		
Girz, 2013	AN, BN, EDNOS	Canada	FBT	17		30.0	57.0	61.7	100.0	16.1	52.9	52.9
Goldstein, 2011	AN, EDNOS	Australia	CBT	26		15.0		49.3	100.0	15.1	28.5	17.9
Goldstein, 2016	AN, EDNOS	Australia	FBT	37	22.7	12.0			91.9	15.6		54.1
Gowers, 2010*	AN	UK	CBT	55	0.0	13.0		23.8	93.0	15.1		
Henderson, 2014	AN, BN, EDNOS	Canada	FBT	65				70.4	100.0	15.0		
Hiney-Saunders, 2021	AN	UK	CBT	48				66.7	100.0	14.9		
Hollesen, 2013	AN, atypical AN	Denmark	MFT	20				48.3	100.0	14.9		50.0
Hughes, 2014	AN	Australia	FBT	35					85.7	15.3		
Hughes, 2017	AN	Australia	FBT	42			26.0	48.3	88.1	15.3	7.1	33.3
Hurst, 2019	AN	Australia	FBT + CBT-P	21	19.0			16.5	100.0	14.9		
Le Grange, 2016*	AN	Australia	FBT	55	0.0	11.0	37.0	36.7	89.1	15.4	10.9	21.8
Le Grange, 2016*	AN	Australia	PFT	52	0.0	10.0	37.0	35.0	86.3	15.7	3.9	29.4
Le Grange, 2021	AN	Australia	FBT	82	4.4	10.6	25.6	41.7	91.5	15.1		45.1
Le Grange, 2022	AN, BN, BED	USA	FBT	51	9.3	10.3	53.5	28.3	78.4	15.7		47.0
Le Grange, 2022	AN, BN, BED	USA	CBT-E	46	9.3	24.4	53.5	53.3	87.0	15.6		67.4
Lebow, 2021a	AN, O, ARFID	USA	FBT	15	6.3		0.0		60.0	15.5	26.7	73.3
Lebow, 2021b	AN	USA	FBT	15		10.3	13.3		86.7	15.7	0.0	66.0
Lim, 2023	AN, BN, O	Australia	FBT	78					84.6	14.2		60.3
Madden, 2015*	AN	Australia	FBT (MS)	41	3.5	7.4	6.1	50.0	95.1	14.9		34.2
Madden, 2015*	AN	Australia	FBT (WR)	41	3.5	7.9	6.1	53.3	95.1	14.9		43.9
Rosling, 2016	AN	Sweden	FBT	29	0.0	9.1		38.1	100.0	15.1	8.5	
Rosling, 2016	EDNOSr	Sweden	FBT	112	0.0	12.0		38.1	100.0	15.2	8.5	
Schlegl, 2015	AN	Germany	CBT	238	0.0	24.7	80.3	72.1	100.0	15.7	23.1	68.1
Schmidt, 2007*	BN, EDNOS	UK	FBT	41	14.1	31.2			100.0	17.9	34.0	63.0
Schmidt, 2007*	BN, EDNOS	UK	GSH-CBT	44	14.1	30.0			95.5	17.4	34.0	68.2
Simic, 2022	AN	UK	FBT	290	0.0	12.6		50.0	93.0	14.8	38.3	55.9
Simic, 2022	BN	UK	FBT/CBT	67	0.0	18.3			93.0	14.8	20.9	55.9
Spettigue, 2019	AN-R	Canada	FBT	117				53.3	100.0	15.1		
Spettigue, 2019	AN-BP	Canada	FBT	36				73.3	100.0	15.8		
Terache, 2023	AN	Belgium	MFT	150				53.3	96.0	15.6		
Thompson, 2020	AN	Australia	FBT	61					100.0	14.7		
Van Huysse, 2022	AN, atypical AN	USA	FBT	98		10.9	77.6		92.9	15.1		
Zanna, 2017	AN, EDNOSr	Italy	FBT	60		14.0	38.3	74.5	93.3	14.8	73.4	

*Note* Empty cells mean that the information on this variable was not provided. Study: Roman letters in parentheses indicate different subgroups within the same study. * = RCT. Disorder: AN = Anorexia Nervosa, ARFID = Avoidant Restrictive Food Intake Disorder, BED = Binge Eating Disorder, BN = Bulimina Nervosa, BP = Binge/purge, EDNOS = Eating disorder not otherwise specified, O = other specified feeding and eating disorder, r = restrictive type. Treatment: CBT = Cognitive Behavior Therapy, CBT-E = Cognitive Behavior Therapy-Enhanced, CBT-P = Cognitive Behavior Therapy-Perfectionism, FBT = Family Based Treatment, FGP = Family Group Psychoeducation, GSH = Guided Self-Help, MFT = Multifamily Therapy, MS = medical stabilization, PFT = Parent-Focused Treatment, WR = weight restoration. Declining = proportion that fulfilled inclusion criteria, offered treatment, but declined it. Dur. = duration of the eating disorder. Previous Tx = proportion that previously had received treatment for their ED. Severity = the mean of the sample on the ED-measure divided by the maximun score possible for that measure. Medicated = proportion taking a prescribed antidepressant medication at the inclusion in the study. Comorbidity = proportion of the sample that fulfilled criteria for any psychiatric disorder at inclusion of the study

#### Treatment data

The treatment data are presented in Table 2. The treatment setting was outpatient care in 37 conditions, daycare in five, daycare followed by outpatient care in one, inpatient care in five, inpatient followed by daycare in one, and inpatient followed by outpatient care in four conditions. Treatment format was family in 24 conditions, individual in nine, a combination of formats in 19, and parent only treatment in one. The treatment was carried out over a mean of 7.1 months (SD 3.5; range 1.5–13.4) and calculated as hours of treatment the mean was 35.3 (SD 41.0; range 6–180). Follow-up assessment was done in 24 conditions (45%) and on average 17.7 months (SD 22.9; range 5–83) after the end of treatment. ITT statistical analysis was provided for 38 conditions (72%) and completer analysis for 15.

**Table 2:** Treatment data for the included studies

Study	Disorder	Subgroup	Tx setting	Tx format	# of therapists	Tx hours	Tx months	% attr.	Fup months	Stat. Analysis
Accurso, 2018	AN	FBT	O	F	7	19.0	5.0	0.0		ITT
Anastasiadou, 2020	AN, BN, BED, O	CBT + App	O (D)	I	7	27.8	3.0	29.2		ITT
Anderson, 2017	AN	FBT Tele	O	F	1	18.4	6.0	0.0	6	ITT
Bentz, 2021	AN	FBT	O	F	Multiple	20.0	12.0	7.6		Compl
Calugi, 2019	AN	CBT-E	D + O	I + G	Multiple	104.0	5.0	9.7	12	ITT
Chew, 2021	AN	FBT	O	F	2	10.6	7.0	15.0	17	ITT
Coelho, 2019	AN	FBT	O	F	5	20.0	7.0	9.7		ITT
Couturier, 2010	AN	FBT	O	F	3	13.0	12.0	14.3		ITT
Craig, 2019	AN, BN	CBT-E	O	I	1	14.2		38.9		ITT
Dalle Grave, 2014	AN	CBT-E	I	I	Multiple	104.0	5.0	3.7	12	Compl
Dalle Grave, 2015	BN, BED, EDNOS	CBT-E	O	I	3	20.0	5.0	25.0		ITT
Dalle Grave, 2019	AN	CBT-E	O	I + F	3	40.0	10.0	28.6	6	ITT
Dalle Grave, 2020	AN	CBT-E	I + D	I	Multiple	104.0	5.0	14.9	14	ITT
Dalle Grave, 2023	AN	CBT-E	O	I	3	40.0	5.0	27.9	5	ITT
Eisler, 2016	AN, EDNOS	FBT	O	F	Multiple	19.0	12.0	11.0	6	ITT
Eisler, 2016	AN, EDNOS	MFT	O	F	Multiple	18.5	12.0	10.6	6	ITT
Gabel, 2014	AN	MFT	O	F				0.0		ITT
Geist, 2000	AN, EDNOS	FBT	I + O	F	3	6.0	4.0	0.0		ITT
Geist, 2000	AN, EDNOS	FGP	I + O	F	3	12.0	4.0	0.0		ITT
Gelin, 2015	AN, BN	MFT	O	F	6	126.0	9.9	8.5		ITT
Girz, 2013	AN, BN, EDNOS	FBT	D	F			6.0			Compl
Goldstein, 2011	AN, EDNOS	CBT	D	C	3	180.0	2.5	7.1	6	Compl
Goldstein, 2016	AN, EDNOS	FBT	O	F	5	14.1	12.0	27.8		ITT
Gowers, 2010	AN	CBT	O	I		22.0	6.0	25.5	60	ITT
Henderson, 2014	AN, BN, EDNOS	FBT	D	F + I	Multiple		3.7	7.7	6	Compl
Hiney-Saunders, 2021	AN	CBT	I	C			4.0	11.1		ITT
Hollesen, 2013	AN, atypical AN	MFT	O	F + I	8	19.0	10.0	37.5		Compl
Hughes, 2014	AN	FBT	O	F			7.6	17.1		Compl
Hughes, 2017	AN	FBT	O	F		13.8	5.0	28.6		Compl
Hurst, 2019	AN	FBT + CBT-P	O	F	2	32.0	12.0	9.5		ITT
Le Grange, 2016	AN	FBT	O	F	8	18.0	6.0	16.4	12	ITT
Le Grange, 2016	AN	PFT	O	P	8	18.0	6.0	13.7	12	ITT
Le Grange, 2021	AN	FBT	O	F		17.0	6.0	8.5	6	ITT
Le Grange, 2022	AN, BN, BED	CBT-E	O	F + I		30.0	9.0	37.0	12	ITT
Le Grange, 2022	AN, BN, BED	FBT	O	F + I		20.0	6.0	35.3	12	ITT
Lebow, 2021a	AN, O, ARFID	FBT	O	F	3	9.2	3.0	13.3		Compl
Lebow, 2021b	AN	FBT	O	F	1	11.1	6.5	20.0		Compl
Lim, 2023	AN, BN, O	FBT	O	F	Multiple	20.0	12.0			ITT
Madden, 2015	AN	FBT (MS)	I + O	C	4	20.0	12.0	10.0	12	ITT
Madden, 2015	AN	FBT (WR)	I + O	C	4	20.0	12.0	13.2	12	ITT
Rosling, 2016	AN	FBT	O (D, I)	C				6.4		Compl
Rosling, 2016	EDNOSr	FBT	O (D, I)	C				18.2		Compl
Schlegl, 2015	AN	CBT	I	C			2.6	19.7		ITT
Schmidt, 2007	BN, EDNOS	FBT	O	C	23	13.0	6.0	12.1	6	ITT
Schmidt, 2007	BN, EDNOS	GSH-CBT	O	C	23	13.0	6.0	18.4	6	ITT
Simic, 2022	AN	FBT	O (D, I)	C		25.0	11.0	9.7	83	ITT
Simic, 2022	BN	FBT/CBT	O (D, I)	C		25.0	7.0	9.7	83	ITT
Spettigue, 2019	AN-R	FBT	I	C			1.5			Compl
Spettigue, 2019	AN-BP	FBT	I	C			1.6			Compl
Terache, 2023	AN	MFT	O	F	Multiple		12.0	6.0	12	Compl
Thompson, 2020	AN	FBT	O (I)	F	2	20.0	13.4			ITT
Van Huysse, 2022	AN, atypical AN	FBT	D	C		150.0	1.2	11.2		ITT
Zanna, 2017	AN, EDNOSr	FBT	D	C			8.0	0.0		ITT

*Note* Empty cells mean that the information on this variable was not provided. Study: Roman letters in parentheses indicate different subgroups within the same study. Disorder: AN = Anorexia Nervosa, ARFID = Avoidant Restrictive Food Intake Disorder, BED = Binge Eating Disorder, BN = Bulimina Nervosa, BP = Binge/purge, EDNOS = Eating disorder not otherwise specified, Ot = other specified feeding and eating disorder, r = restrictive type. Treatment: CBT = Cognitive Behavior Therapy, CBT-E = Cognitive Behavior Therapy-Enhanced, CBT-P = Cognitive Behavior Therapy-Perfectionism, FBT = Family Based Treatment, FGP = Family Group Psychoeducation, GSH = Guided Self-Help, MFT = Multifamily Therapy, MS = medical stabilization, PFT = Parent-Focused Treatment, WR = weight restoration. Tx setting: D = day care hospital, I = inpatient, O = outpatient, D + O = day care followed by outpatient, I + D = inpatient followed by day care, I + O = inpatient followed by outpatient, (D, I) = day care, inpatient were not planned by necessary for some participants. Tx format: C = combination of formats, F = family. G = group, I = individual. # of therapists: Multiple = not specified but more than two. % attr. = proportion who dropped out of those who had at least one session. Statistical analysis: Compl = completers only analysis, ITT = intent to treat analysis

### Methodological data

The research methodology score had a mean of 43.5% (SD 10.8), which corresponds to a raw score of 19.1 points. The RoB classification is presented in SI 5. Among the 12 RCT-conditions 11 had a low and 1 had a moderate RoB. Regarding the 41 NRSI/pre-post conditions 22 had a moderate RoB and 19 had a high RoB.

### Meta-analysis

#### Attrition

Data on attrition were provided for 47 of the conditions (88.7%) and the mean rate was 15.3% (95% CI 12.8–18.2). AN-studies had a mean of 14.0% and Mixed ED-studies 17.8%. The Q between studies (Qb; 1 df) was not significant (1.77, *p* = 0.18).

#### Primary outcome measures

Data on the primary measure of ED-psychopathology were provided for 77% of the conditions, and the results are displayed in Table 3. Sixty-eight percent of the studies that provided such data used the EDE or the EDE-Q, whereas the EDI-2 was used in 22%, and the EDI-3 in 10%. Since they all measure ED-psychopathology pooling within this category was considered to be acceptable. At post-treatment the mean ES across all disorders was large (0.80) and significantly heterogeneous. A subgroup analysis comparing AN and Mixed ED did not yield a significant difference. At follow-up, the mean ES was still large (0.97) and heterogeneous with no significant differences between the ED-disorders. Thus, it seems that the effects of treatment were maintained at follow-up. However, only 45% of the conditions had follow-up data. Regarding publication bias for the ED-psychopathology, Egger’s regression intercept yielded a non-significant *t*-value (0.41, *p* = 0.68). Thus, publication bias does not seem to be a problem for the ED-psychopathology measure.

**Table 3:** Results on ED-psychopathology measures at post and follow-up assessment

Time point	Disorder	*k*	*g*	95% CI	z-value	95% PI	Q-value†	*p*-value
Post	All	41	0.80	0.69–0.91	13.70^a^	0.15; 1.45		
	AN	26	0.85	0.71–0.99	11.65^a^	0.19; 1.51	1.28	0.26
	Mix	15	0.71	0.52–0.90	7.29^a^	0.04; 1.38		
Follow-up	All	19	0.97	0.82–1.12	12.43^a^	0.34; 1.60		
	AN	13	1.05	0.86–1.23	11.35^a^	0.44; 1.65	2.35	0.13
	Mix	6	0.78	0.49–1.07	5.29^a^	0.13; 1.43		

*Note k* = treatment conditions, *g* = Hedges’s g, CI = confidence interval, PI = prediction interval, † = comparison between the disorders

a = *p* < 0.0001

The sensitivity analysis with different pre-post correlation (0.1, 0.3, 0.5, 0.7, and 0.9) yielded the following results at post-treatment; 0.798, 0.799, 0.800, 0.801, and 0.805. Thus, the mean ES changed very little due to the various estimates of the pre-post correlation. Regarding the effect of the various ED-psychopathology measures the overall ES at post was 0.80, when EDI-2 was removed 0.87, and when EDI-3 was removed 0.88. Thus, the overall ES was robust across measures of ED-psychopathology. The method of removing one study at a time was used and the mean ES fell between 0.78 and 0.82, indicating that none of the studies impacted the mean ES unduly.

The results for the primary outcome measure weight in AN are presented in Table 4. Some type of weight measure was provided by all of the conditions with AN-participants and yielded a very large ES (1.64) at post-assessment, which was significantly heterogeneous. The subgroup analysis of the type of weight measure did not show a significant difference between them. At follow-up the ES was even higher (2.07), significantly heterogeneous, and with no significant difference between the measures. The analysis of publication bias yielded a significant Egger’s regression intercept (*t* = 4.21, *p* < 0.001).

**Table 4:** Results on weight measures for AN at post and follow-up assessment

Time point	Measure	*k*	*g*	95% CI	z-value	Q-value	95% PI	Qb†	*p*-value
Post	All	32	1.64	1.37–1.92	11.76^b^	506.3^b^	0.07; 3.22		
	% EBW	17	1.57	1.20–1.95	8.19^b^	265.3^b^	0.01; 3.13	4.62	0.10
	% Mdn BW	8	1.33	0.78–1.88	4.72^b^	116.1^b^	-0.29; 2.94		
	BMI	7	2.18	1.59–2.76	7.31^b^	33.6^b^	0.55; 3.80		
Follow-up	All	13	2.07	1.51–2.62	7.26^b^	196.3^b^	-0.03; 4.16		
	% EBW	8	2.40	1.69–3.11	6.64^b^	89.7^b^	0.13; 4.67	2.24	0.14
	% Mdn BW	5	1.52	0.62–2.43	3.30^a^	94.9^b^	-0.83; 3.88		

*Note k* = treatment conditions, *g* = Hedges’s g, CI = confidence interval, PI = prediction interval, † = comparison between the disorders

a = *p* < 0.001, b = *p* < 0.0001

#### Moderator analyses

Regarding the ED-psychopathology measure, the subgroup analyses of categorical variables are displayed in Table 5, left hand side. Using Holm-Bonferroni correction only the RoB-variable yielded a significant difference (Q = 27.7, *p* = 0.001) between the included categories. Subsequent pair-wise comparisons showed that studies with moderate RoB had significantly higher ES than studies with either high (Q = 19.87, *p* = 0.001) or low RoB (Q = 13.14, *p* = 0.001). Regarding the five continuous variables, the meta-regression analyses yielded a significant point estimate (0.235, *z* = 2.73, *p* = 0.006) for mean age of the sample; studies with higher mean age were associated with a higher ES.

When it comes to the weight measures (Table 5, right hand side) there were two variables significantly moderating the ES. First, the type of statistical analysis showed a significant difference (Q = 10.11, *p* = 0.001), with higher ES for studies using completer analyses (2.21) than those using ITT analysis (1.37). Second, the continent at which the study was carried out also differed significantly (Q = 9.30, *p* = 0.01), and the subsequent pair-wise comparisons showed that studies from Europe had significantly higher ES (2.17; Q = 5.47, *p* = 0.02) than studies from North America (1.40) and studies from Australia (1.33; Q = 9.29, *p* = 0.002). None of the continuous variables acted as a significant moderator of the ES for weight measures.

**Table 5:** Subgroup analyses of categorical variables in ED-psychopathology measures and weight measures in AN at post-treatment

	ED-psychopathology						Weight					
Variable	*k*	*g*	95% CI	Q-value	Qb	*p*-value	*k*	*g*	95% CI	Q-value	Qb	*p*-value
*Design*												
NRSI/Pre-post	31	0.88	0.75-1.01	156.2^d^	6.02	0.014	27	1.67	1.35-2.00	424.4^a^	0.08	0.78
RCT	10	0.56	0.33-0.78	20.2^a^			5	1.55	0.79-2.00	72.7^a^		
*Statistical analysis*												
Intent-to-treat	29	0.88	0.75-1.00	130.3^d^	4.65	0.03	22	1.37	1.08-1.66	325.8^a^	10.11	0.001
Treatment completers	12	0.62	0.43-0.82	25.1^d^			10	2.21	1.78-2.64	23.9^a^		
*Risk of bias*												
High	11	0.56	0.39-0.73	15.4	26.74	0.001	12	1.77	1.29-2.26	157.1^a^	0.39	0.82
Moderate	21	1.05	0.92-1.17	75.2^d^			15	1.59	1.15-2.03	240.8^a^		
Low	9	0.57	0.38-0.77	20.1^b^			5	1.55	0.80-2.31	72.7^a^		
*Treatment format*												
Family	17	0.68	0.49-0.87	59.3^d^	2.86	0.24	16	1.33	0.98-1.68	271.5^a^	5.84	0.05
Individual	9	0.92	0.68-1.16	74.0^d^			5	1.93	1.26-2.60	6.3		
Combined formats	15	0.85	0.66-1.04	46.0^d^			11	1.97	1.53-2.40	100.1^a^		
*Continent*												
Europe	21	0.90	0.76-1.05	132.0^d^	5.22	0.07	13	2.17	1.77-2.58	53.9^a^	9.30	0.010
North America	12	0.59	0.35-0.82	30.6^c^			9	1.40	0.93-1.87	146.3^a^		
Australia	8	0.75	0.50-1.00	8.1			9	1.33	0.87-1.39	120.6^a^		

*Note k* = treatment conditions, *g* = Hedges’s g, CI = confidence interval, *Q* = test of heterogeneity among the studies, † = comparison between the subcategories

a = *p* < 0.05, b = *p* < 0.01, c = *p* < 0.001, d = *p* < 0.0001

#### Secondary outcome measure

Depression was assessed in 40% of the conditions. At post-treatment the mean ES was 0.61 (95% CI 0.47–0.75, *z* = 8.55, *p* = 0.0001) and heterogeneous (Q = 70.1, *p* = 0.0001), and at follow-up it was 0.67 (95% CI 0.53–0.82, *z* = 9.13, *p* = 0.0001) but not heterogeneous. Regarding publication bias, Egger’s regression intercept was not significant (*t* = 1.17, *p* = 0.25).

### Comparison between treatment methods

The results for the comparison between treatment conditions using family therapy for ED and those using CBT are presented in Table 6. On the ED-psychopathology measure the mean ES for CBT (1.05) at post-treatment was significantly higher (*p =* 0.001) than that for family therapy (0.68). At follow-up assessment there was also a higher (*p* = 0.022) ES for CBT (1.20) than for family therapy (0.84). On the weight measures for AN both treatments showed very large ESs at post-treatment and even larger at follow-up assessment, without being significantly different.

**Table 6:** Comparison of CBT and Family therapy (FT) on ED-psychopathology measures and weight (AN only)

Time point	Treatment	*k*	*g*	95% CI	z-value	95% PI	Q-value	*p*-value
*ED-psychopathology*								
Post	CBT	14	1.05	0.87.1.22	11.77^a^	0.48; 1.62	10.87	0.001
	FT’	24	0.68	0.55–0.81	9.99^a^	0.12; 1.24		
Follow-up	CBT	7	1.20	0.96–1.44	9.68^a^	0.63; 1.77	5.28	0.022
	FT’	11	0.84	0.66–1.02	9.06^a^	0.30; 1.39		
*Weight in AN*								
Post	CBT	8	1.94	1.30–2.58	5.95^a^	0.05; 3.83	0.37	0.54
	FT’	21	1.72	1.32–2.10	8.61^a^	-0.11; 3.52		
Follow-up	CBT	5	2.30	1.42–3.18	5.12^a^	-0.05; 4.65	0.02	0.89
	FT’	6	2.22	1.41–3.03	5.36^a^	-0.10; 4.54		

*Note k* = treatment conditions, g = Hedges’s g, CI = confidence interval, PI = prediction interval, † = comparison between the disorders

a = *p* < 0.0001

Family therapy for ED and CBT were compared on the background variables for which data were available from most studies. The mean age of the samples was 15.8 and 15.3 years, the proportion of females was 96.5% and 95.2%, the proportion who declined participation in the studies was 15.8% and 12.5%, the mean duration of the eating disorders was 21.3 and 12.3 months, the POMRS scores were 45.1% and 44.8%, and the mean severity on the ED-psychopathology measure was 55.3% and 46.9% for CBT and family therapy, respectively. None of the differences between the treatments were significant. Regarding categorical variables there was no significant difference on number of RCTs versus open trials (*p* = 1.0), ITT vs. completer statistical analysis (*p* = 0.29), low vs. moderate/high RoB (*p* = 0.23), and inpatient vs. outpatient care (*p* = 0.71). However, CBT -studies were done in Europe (84.6%) to a larger extent than in other continents, whereas family therapy-studies were carried out in North America (39.3%), Europe (35.7%), and Australia (25%).

### Effectiveness-efficacy comparison

#### Background and treatment variables

The comparisons of effectiveness and efficacy studies on some background and treatment variables are displayed in Table 7. Applying the Holm-Bonferroni correction on the 7 *t*-tests in this table yielded no significant difference between the two types of studies. This makes for a fair comparison regarding effect sizes.

**Table 7:** Some background and treatment data (M and SD) for effectiveness and efficacy studies

Type of study	k	Mean age	% females	% severity	% comorbid	% medicated	Tx hours	% attrition
Effectiveness	53	15.4 (0.8)	94.1 (7.4)	49.3 (13.3)	42.9 (19.7)	27.4 (22.3)	35.3 (41.0)	15.3 (10.6)
Efficacy	20	15.3 (1.9)	88.7 (12.8)	45.3 (15.2)	46.5 (15.5)	12.4 (12.6)	21.8 (16.2)	18.7 (9.7)
*p*-value		0.75	0.03	0.31	0.56	0.03	0.18	0.16

#### Effect size on primary outcome measure

The comparison between effectiveness and efficacy studies on eating psychopathology and weight are presented in Table 8. On the ED-psychopathology measure at post-treatment there were large ESs for both types of studies with a small difference between them (0.80 vs. 0.84) with all disorders combined. Regarding AN there was a tendency for effectiveness studies to yield a higher ES than efficacy studies (0.85 vs. 0.63), and for the Other category there was a tendency that efficacy studies gave a higher ES than effectiveness studies (1.16 vs. 0.72). However, when applying the Holm-Bonferroni correction none of these differences was significant. At follow-up assessment for both types of studies the mean ES across disorders was maintained with a small difference between them (0.97 vs. 1.09). For the individual disorders there was no significant difference between the types of studies in AN, but for Other disorders efficacy studies yielded a significantly higher ES than effectiveness studies. However, this difference must be interpreted with caution since there were only three efficacy studies.

The results for weight in AN-conditions are shown in the lower part of Table 8. There were very large ESs for both types of studies at post-treatment and even higher at follow-up assessment. However, the difference between them was not significant.

**Table 8:** Effect sizes on ED-psychopathology measures and weight (for AN only) for effectiveness and efficacy studies at post-assessment and follow-up assessment

Time point	Disorder	Study type	*k*	*g*	95% CI	z-value	95% PI	Qb†	*p*-value
*ED-psychopathology*									
Post	All disorders	Effectiveness	41	0.80	0.68–0.92	13.21^a^	0.13; 1.48	0.10	0.76
		Efficacy	18	0.84	0.65–1.02	8.65^a^	0.14; 1.53		
	AN	Effectiveness	26	0.85	0.73–0.97	24.12^a^	0.35; 1.35	3.55	0.06
		Efficacy	12	0.63	0.44–0.82	6.52^a^	0.11; 1.60		
	Other*	Effectiveness	15	0.72	0.46–0.95	6.02^a^	-0.18; 1.61	3.90	0.05
		Efficacy	6	1.16	0.79–1.54	6.07^a^	0.22; 2.10		
Follow-up	All disorders	Effectiveness	19	0.97	0.82–1.12	12.43^a^	0.34; 1.60	0.46	0.50
		Efficacy	11	1.09	0.84–1.35	8.26^a^	0.32; 1.87		
	AN	Effectiveness	13	1.04	0.88–1.20	12.46^a^	0.52; 1.56	2.97	0.09
		Efficacy	8	0.79	0.55–1.02	6.47^a^	0.23; 1.34		
	Other*	Effectiveness	6	0.79	0.48–1.09	5.09^a^	0.06; 1.57	13.52	0.001
		Efficacy	3	1.77	1.34–2.20	8.13^a^	0.91; 2.63		
*Weight*									
Post	AN	Effectiveness	32	1.64	1.37–1.91	11.87^a^	0.14; 3.14	1.83	0.18
		Efficacy	12	2.02	1.54–2.50	8.31^a^	0.47; 3.57		
Follow-up	AN	Effectiveness	13	2.07	1.51–2.62	7.26^a^	0.17; 3.92	0.82	0.37
		Efficacy	8	2.44	1.76–2.12	7.06^a^	0.50; 4.38		

*Note k* = treatment conditions, g = Hedges’s g, CI = confidence interval, PI = prediction interval, † = comparison between the disorders

*Other is the combination of BN, BED, and Mixed disorders

a = *p* < 0.0001

## Discussion

The current meta-analysis aimed to investigate how family therapy and CBT work in the treatment of ED in children and adolescents when delivered in routine clinical care. The first aim was to examine the effectiveness of ED focused family therapy and CBT for ED in children and adolescents. Across the various measures of ED-psychopathology and weight measures for AN, the overall within-group effect size was large to very large for all disorders combined at post, with no difference between the AN and mixed ED group. Direct comparisons of the effect size estimates to the outcomes reported in the evidence-based updates on psychosocial treatments for ED in children and adolescents [10, 78] and in systematic reviews [22, 79, 80] are challenged by the use of various outcome measures across the included studies. However, among specific studies that have reported similar effect size calculations of ED-psychopathology and weight measures for AN, our finding of large to very large ES) compares favorably to the ES reported (but not pooled) by Datta et al., [10].

The overall attrition rate across the studies was only 15%, with no difference between the AN-studies and Mixed ED-studies. This figure is comparable with the reported dropout rate in adolescent RCTs for AN that fall between 10 and 20% [81], but considerably lower than the 50% reported for adolescents being treated for AN in a recent review of psychotherapies for ED [82]. As dropout from treatments for ED is reported to be a significant problem, the results indicate that family therapy and CBT for ED were acceptable to youth and their caregivers. To broaden the view, outcome data for the common comorbidity of depression was extracted. However, only 40% of the conditions provided these data, and although a moderate effects size was found the results may not be representative for the entire body of studies.

The second aim was to evaluate methodological quality and RoB in the effectiveness studies and examine potential moderators of treatment outcome. The result of the methodological quality assessment was encouraging given the high proportion of open trials and is comparable to recent meta-analyses on the effectiveness of evidence-based treatments for externalizing disorders [35], and autism spectrum disorders [36], but somewhat lower compared to internalizing disorders in children [32]. Methodological flaws were noted in several of the studies, with RCTs having a lower RoB. Overall, results showed that the majority of the studies had a moderate or high RoB.

Characteristics of the patient sample and study variables were examined as potential moderators influencing treatment outcome. Moderator analyses of the categorical variables did not provide support for a difference in ESs between RCTs and NRSI/pre-post studies across the outcomes of ED-psychopathology measures and weight measures in AN at post-treatment. Furthermore, treatment format did not moderate outcome. These findings corroborate the results from three previous meta-analyses on the effectiveness of evidence-based treatments for internalizing, externalizing, and autism spectrum disorders in children and adolescents [32, 35, 36]. Moderator analyses also showed that studies with moderate RoB produced larger effects on the ED psychopathology outcome measure compared to studies with high or low RoB. This result is similar to the finding in a meta-analysis of CBT effectiveness studies in adult ED [48]. There was also a difference in ES of weight measures outcome in AN between continents, with studies conducted in Europe reporting a higher ES compared to those from North America and Australia. Similar results were found for internalizing disorders in children [32] and obsessive compulsive disorder in adults [44]. For type of statistical analysis, the use of treatment completers data moderated the weight measures outcome in AN. The use of completer analyses may inflate results of treatment, as it may be that patients who drop out from treatment more often are not benefitting or find the treatment unacceptable [83]. It is therefore encouraging that 70% of the studies reported ITT data.

Of the continuous variables age moderated the outcome of ED-psychopathology. The finding that higher mean age was associated with a higher ES was in line with the meta-analyses by Svaldi [46] and van der Berg [38]. Although it has not been possible to draw firm conclusion regarding moderators of treatment outcome for ED in children and adolescents [84], one study found older age to negatively impact outcome [85], whereas another found age not being related to change in ED-symptoms [86]. As such, our results regarding age need to be interpreted with caution. Moderator analyses did not provide support for the other continuous variables to moderate ED treatment effects.

The third aim was to compare the effectiveness of family therapy and CBT for ED when delivered in routine clinical care. There was a difference on the ED-psychopathology measure in favor of CBT with a large compared to a moderate ES at post treatment. At follow-up the difference between CBT and family therapy remained with a very large compared to a large ES. No significant differences between family therapy for ED and CBT were found for the weight outcome in AN. Family therapy for ED and CBT studies were compared on 11 background and treatment variables. The only variable that showed a significant difference was the proportion of family therapy and CBT studies carried out in different continents, where CBT-studies more often were conducted in Europe.

Whereas family therapy for ED has the strongest evidence-base for children and adolescents with ED, no therapy is effective for everyone. Data suggest that the best evidence-based approach for adolescent ED leaves about 50% of the patients not fully remitted following treatment [87]. To the best of our knowledge, there are only three RCTs comparing CBT and family therapy for ED in adolescents. Ball and Mitchell [88] found no significant differences on EDE or BMI in AN-patients, Schmidt et al., [89] studied BN-patients and reported a significantly larger reduction of binge eating for guided self-help CBT than family therapy for ED at post-treatment but no difference at follow-up, and Le Grange et al., [90], also working with BN, found a significant difference in abstinence rate in favor of family therapy for ED, which disappeared at follow-up. Thus, these RCTs do not show higher effect for either of these treatments and more RCTs comparing different treatments are warranted.

Our findings do not address the issue of one of the treatments’ superiority over the other for children and adolescents with ED, or for whom these therapies may be most suitable. The therapies differ in their conceptualizations, levels of parental involvement, in strategies, procedures, and postulated mechanism of action adopted to produce the change. For the CBT studies the treatment format was individual in 54% of the studies and a combination of individual and family or group in 46%. In comparison, family involvement has been the corner stone in all studies of family therapy for ED (which was combined with individual treatment in 39% of the studies). Taken together, our results lend support for the use of CBT for ED among children and adolescents and that it may be considered an option when treating children and adolescents with ED in routine clinical care, and not only when family therapy for ED fails or is not feasible.

The fourth aim was to compare the outcomes of family therapy and CBT for ED when delivered in routine clinical care to efficacy studies for the same disorders. As an initial step, effectiveness and efficacy studies were compared on relevant background and treatment variables, and no differences were found. The effectiveness studies of the ED combined, AN, or the Other ED generated post treatment ES for ED-psychopathology outcomes that were similar to the ES from efficacy studies. The ES in both settings were in the large to very large range at post and follow-up assessments. The only difference between the studies was for the Other ED group at follow-up, where the efficacy studies produced a very large compared to a large ES in the effectiveness studies. However, only three efficacy conditions could be included in this comparison. For the weight measure outcome in the AN-studies the ES at post and follow-up were in the very large range with no differences between the efficacy and effectiveness studies. This pattern of findings with no significant differences in outcomes across efficacy and effectiveness studies replicates the findings in other meta-analyses of evidence-based treatments for various mental health disorders in children [32, 35, 36], and adults [44, 48, 73–75].

Strong methodological elements of the current meta-analysis included a power analysis indicating a high power to detect a small effect size. Furthermore, pairs of researchers screened abstracts and extracted information from the included studies with disparities solved in consensus, and ratings of methodological quality and RoB were done by one of the authors and independently by another.

### Study limitations and future directions

Although our classification criteria of effectiveness studies were predefined and assessment could be made reliably by trained raters, studies differed on the quality of reporting the needed information. Thus, judgment was based on the sometimes limited and ambiguous information available, and perhaps some studies are missed that should have been included. This meta-analysis attempted to include studies on all EDs, but for BED, OSFED, and ARFID, however, there were few or no clinical trials to be included, limiting the generalizability of our findings for these disorders. Furthermore, as only five studies included children younger than 11 years the age range should be considered when interpreting the findings. Also, pooling the outcome based on different measures of ED-psychopathology and weight measure for AN is a limitation and international consensus in the assessment of ED is needed. However, the sensitivity analysis of ED psychopathology measures and sub-group analysis of weight measures showed that the different measures did not influence the pooled ES significantly. Finally, the majority of the studies had a moderate or high RoB.

There are several areas in which the field of ED in children and adolescents can be improved. The lack of consensus definitions of response and remission in the assessment of ED calls for effort to establish such standards that can be applied consistently across studies. Furthermore, reporting of outcome separately for each diagnostic group in studies with transdiagnostic samples data and at long-term follow-up assessments is recommended.

## Conclusion

Our findings support the effectiveness of family therapy and CBT for ED in children and adolescents. Adequately trained clinicians who provide these treatments in their work with children and adolescents and their families in routine clinical care can achieve outcomes comparable to those in research clinic settings, and the treatments have a low attrition rate. Whereas family therapy for ED has the strongest evidence-base, our results suggest that CBT could be considered an option when treating children and adolescents with ED in routine clinical care. At the same time, the results also suggest there is room for improvement as a substantial number of children and adolescents with ED do not respond to the treatments currently available.

## Electronic supplementary material

Below is the link to the electronic supplementary material.

Supplementary Material 1

## Data Availability

No datasets were generated or analysed during the current study.

## References

[1] American Psychiatric Association (2013) Diagnostic and statistical manual of mental disorders: DSM-5, 5th edn. American Psychiatric Association, Washington, D.C.

[2] Swanson SA, Crow SJ, Le Grange D, Swendsen J, Merikangas KR (2011) Prevalence and correlates of eating disorders in adolescents: results from the national comorbidity survey replication adolescent supplement. Arch Gen Psychiat 68(7):714–723. 10.1001/archgenpsychiatry.2011.22 21383252 10.1001/archgenpsychiatry.2011.22 PMC5546800

[3] Udo T, Grilo CM (2018) Prevalence and correlates of DSM-5–defined eating disorders in a nationally representative sample of US adults. Biol Psychiat 84(5):345–354 29859631 10.1016/j.biopsych.2018.03.014 PMC6097933

[4] Smink FR, Van Hoeken D, Hoek HW (2012) Epidemiology of eating disorders: incidence, prevalence and mortality rates. Curr Psychiatry Rep 14(4):406–414. 10.1007/s11920-012-0282-y 22644309 10.1007/s11920-012-0282-y PMC3409365

[5] López-Gil JF, García-Hermoso A, Smith L, Firth J, Trott M, Mesas AE et al (2023) Global proportion of disordered eating in children and adolescents: a systematic review and meta-analysis. JAMA Pediatr 177(4):363–372. 10.1001/jamapediatrics.2022.5848 36806880 10.1001/jamapediatrics.2022.5848 PMC9941974

[6] Madigan S, Vaillancourt T, Dimitropoulos G, Premji S, Kahlert SM, Zumwalt K et al (2024) A systematic review and Meta-analysis: child and adolescent Healthcare utilization for eating disorders during the COVID-19 pandemic. J Am Acad Child Adolesc Psychiatry. 10.1016/j.jaac.2024.02.009 38431196 10.1016/j.jaac.2024.02.009

[7] Auger N, Potter BJ, Ukah UV, Low N, Israël M, Steiger H et al (2021) Anorexia nervosa and the long-term risk of mortality in women. World Psychiatry 20(3):448. 10.1002/wps.20904 34505367 10.1002/wps.20904 PMC8429328

[8] Nielsen S, Vilmar JW (2021) What can we learn about eating disorder mortality from eating disorder diagnoses at initial assessment? A Danish nationwide register follow-up study using record linkage, encompassing 45 years (1970–2014). Psychiatry Res 303:114091 34246009 10.1016/j.psychres.2021.114091

[9] Hambleton A, Pepin G, Le A, Maloney D, Touyz S, Maguire S (2022) Psychiatric and medical comorbidities of eating disorders: findings from a rapid review of the literature. J Eat Disord 10(1):132. 10.1186/s40337-022-00654-2 36064606 10.1186/s40337-022-00654-2 PMC9442924

[10] Datta N, Matheson BE, Citron K, Van Wye EM, Lock JD Evidence based update on psychosocial treatments for eating disorders in children and adolescents. J Clin Child Adolesc Psychol 52(2):159–170. 10.1080/15374416.2022.2109650 10.1080/15374416.2022.2109650 35950931

[11] Crone C, Fochtmann LJ, Attia E, Boland R, Escobar J, Fornari V et al (2023) The American Psychiatric Association practice guideline for the treatment of patients with eating disorders. Am J Psychiatry 180(2):167–171. 10.1176/appi.ajp.23180001 36722117 10.1176/appi.ajp.23180001

[12] National Institute for Health and Care Excellence (2017) Eating disorders: recognition and treatment: NICE. https://www.nice.org.uk/guidance/ng69 28654225

[13] Hay P, Chinn D, Forbes D, Madden S, Newton R, Sugenor L et al (2014) Royal Australian and New Zealand College of Psychiatrists clinical practice guidelines for the treatment of eating disorders. Aust N Z J Psychiatry 48(11):977–1008. 10.1177/0004867414555814 25351912 10.1177/0004867414555814

[14] Couturier J, Isserlin L, Norris M, Spettigue W, Brouwers M, Kimber M et al (2020) Canadian practice guidelines for the treatment of children and adolescents with eating disorders. J Eat Disord 8(1):1–80. 10.1186/s40337-020-0277-8 32021688 10.1186/s40337-020-0277-8 PMC6995106

[15] Hilbert A, Hoek HW, Schmidt R (2017) Evidence-based clinical guidelines for eating disorders: international comparison. Curr Opin Psychiatr 30(6):423. 10.1097/YCO.0000000000000360 10.1097/YCO.0000000000000360 PMC5690314 28777107

[16] Eisler I, Simic M, Hodsoll J, Asen E, Berelowitz M, Connan F et al (2016) A pragmatic randomised multi-centre trial of multifamily and single family therapy for adolescent anorexia nervosa. BMC Psychiatry 16(1):1–14. 10.1186/s12888-016-1129-6 27881106 10.1186/s12888-016-1129-6 PMC5122159

[17] Lock J, Le Grange D (2015) Treatment manual for anorexia nervosa: a family-based approach. Guilford 10.1111/j.1475-3588.2004.00104_7.x 32797535

[18] Le Grange D (1999) Family therapy for adolescent anorexia nervosa. J Clin Psychol 55(6):727–739. 10.1002/(SICI)1097-4679(199906) 10445863 10.1002/(sici)1097-4679(199906)55:6<727::aid-jclp6>3.0.co;2-3

[19] Le Grange D, Gorrell S, Hughes EK, Accurso EC, Yeo M, Pradel M, Sawyer SM (2020) Delivery of family-based treatment for adolescent anorexia nervosa in a public health care setting: research versus non-research specialty care. Front Psychiatry 10:1001. 10.3389/fpsyt.2019.01001 32038332 10.3389/fpsyt.2019.01001 PMC6987240

[20] Freizinger M, Jhe G, Pluhar E, Mancini L (2021) Integrating family-based treatment principles in the acute inpatient treatment of adolescents with restrictive eating disorders. Psychol Res Behav Manag 14:449–454. 10.2147/PRBM.S304921 33859508 10.2147/PRBM.S304921 PMC8044072

[21] Baudinet J, Eisler I, Dawson L, Simic M, Schmidt U (2021) Multi-family therapy for eating disorders: a systematic scoping review of the quantitative and qualitative findings. Int J Eat Disord 54(12):2095–2120. 10.1002/eat.23616 34672007 10.1002/eat.23616 PMC9298280

[22] Dalle Grave R, Conti M, Sartirana M, Sermattei S, Calugi S Enhanced cognitive behaviour therapy for adolescents with eating disorders: a systematic review of current status and future perspectives. Ital J Eat Disord Obes (3):1–11. 10.32044/ijedo.2021.01

[23] Fairburn C (1981) A cognitive behavioural approach to the treatment of bulimia. Psychol Med 11(4):707–711. 10.1017/s0033291700041209 6948316 10.1017/s0033291700041209

[24] Agras WS, Bohon C (2021) Cognitive behavioral therapy for the eating disorders. Annu Rev Clin Psychol 17:417–438. 10.1146/annurev-clinpsy-081219-110907 33962536 10.1146/annurev-clinpsy-081219-110907

[25] Fairburn CG, Cooper Z, Shafran R (2003) Cognitive behaviour therapy for eating disorders: a transdiagnostic theory and treatment. Behav Res Ther 41(5):509–528. 10.1016/s0005-7967(02)00088-8 12711261 10.1016/s0005-7967(02)00088-8

[26] Fairburn CG (2008) Cognitive behavior therapy and eating disorders. Guilford Press

[27] Dalle Grave R, Calugi S, Doll HA, Fairburn CG (2013) Enhanced cognitive behaviour therapy for adolescents with anorexia nervosa: an alternative to family therapy? Behav Res Ther 51(1):R9–R12. 10.1016/j.brat.2012.09.008 23123081 10.1016/j.brat.2012.09.008 PMC3662031

[28] Dalle Grave R, Calugi S (2020) Cognitive behavior therapy for adolescents with eating disorders. Guilford 10.1186/s13030-024-00315-7 PMC11373333 39232751

[29] Dalle Grave R, Calugi S, Conti M, Doll H, Fairburn CG (2013) Inpatient cognitive behaviour therapy for anorexia nervosa: a randomized controlled trial. Psychother Psychosom 82(6):390–398. 10.1159/000350058 24060628 10.1159/000350058 PMC3884188

[30] Weisz JR, Jensen-Doss A, Hawley KM (2006) Evidence-based youth psychotherapies versus usual clinical care: a meta-analysis of direct comparisons. Am Psychol 61(7):671. 10.1037/0003-066X.61.7.671 17032068 10.1037/0003-066X.61.7.671

[31] Stewart RE, Chambless DL (2009) Cognitive-behavioral therapy for adult anxiety disorders in clinical practice: a meta-analysis of effectiveness studies. J Consult Clin Psychol 77(4):595–606. 10.1037/a0016032 19634954 10.1037/a0016032 PMC8022196

[32] Wergeland GJH, Riise EN, Öst LG (2021) Cognitive behavior therapy for internalizing disorders in children and adolescents in routine clinical care: a systematic review and meta-analysis. Clin Psychol Rev 83:101918. 10.1016/j.cpr.2020.101918 33186776 10.1016/j.cpr.2020.101918

[33] Bentz M, Pedersen SH, Moslet U (2021) An evaluation of family-based treatment for restrictive-type eating disorders, delivered as standard care in a public mental health service. J Eat Disord 9:1–12. 10.1186/s40337-021-00498-2 34715920 10.1186/s40337-021-00498-2 PMC8555240

[34] Golden NH, Katzman DK, Sawyer SM, Ornstein RM, Rome ES, Garber AK et al (2015) Update on the medical management of eating disorders in adolescents. J Adolesc Health 56(4):370–375. 10.1016/j.jadohealth.2014.11.020 25659201 10.1016/j.jadohealth.2014.11.020

[35] Riise EN, Wergeland GJH, Njardvik U, Öst LG (2021) Cognitive behavior therapy for externalizing disorders in children and adolescents in routine clinical care: a systematic review and meta-analysis. Clin Psychol Rev 83:101954. 10.1016/j.cpr.2020.101954 33418192 10.1016/j.cpr.2020.101954

[36] Wergeland GJH, Posserud M-B, Fjermestad K, Njardvik U, Öst L-G (2022) Early behavioral interventions for children and adolescents with autism spectrum disorder in routine clinical care: a systematic review and meta-analysis. Clin Psychol Sci Pract 29(4):400

[37] Hilbert A, Petroff D, Herpertz S, Pietrowsky R, Tuschen-Caffier B, Vocks S, Schmidt R (2020) Meta‐analysis on the long‐term effectiveness of psychological and medical treatments for binge‐eating disorder. Int J Eat Disord 53(9):1353–1376. 10.1002/eat.23297 32583527 10.1002/eat.23297

[38] van den Berg E, Houtzager L, de Vos J, Daemen I, Katsaragaki G, Karyotaki E et al (2019) Meta-analysis on the efficacy of psychological treatments for anorexia nervosa. Eur Eat Disord Rev 27(4):331–351. 10.1002/erv.2683 31124215 10.1002/erv.2683

[39] Hilbert A, Petroff D, Herpertz S, Pietrowsky R, Tuschen-Caffier B, Vocks S, Schmidt R (2019) Meta-analysis of the efficacy of psychological and medical treatments for binge-eating disorder. J Consult Clin Psychol 87(1):91. 10.1037/ccp0000358 30570304 10.1037/ccp0000358

[40] Murray SB, Quintana DS, Loeb KL, Griffiths S, Le Grange D (2019) Treatment outcomes for anorexia nervosa: a systematic review and meta-analysis of randomized controlled trials. Psychol Med 49(4):535–544. 10.1017/S0033291718002088 30101734 10.1017/S0033291718002088

[41] Davey E, Bennett SD, Bryant-Waugh R, Micali N, Takeda A, Alexandrou A, Shafran R (2023) Low intensity psychological interventions for the treatment of feeding and eating disorders: a systematic review and meta-analysis. J Eat Disord 11(1):56. 10.1186/s40337-023-00775-2 37016447 10.1186/s40337-023-00775-2 PMC10072817

[42] Linardon J, Wade TD (2018) How many individuals achieve symptom abstinence following psychological treatments for bulimia nervosa? A meta-analytic review. Int J Eat Disord 51(4):287–294. 10.1002/eat.22838 29417609 10.1002/eat.22838

[43] Cuijpers P, Sijbrandij M, Koole SL, Andersson G, Beekman AT, Reynolds CF III (2013) The efficacy of psychotherapy and pharmacotherapy in treating depressive and anxiety disorders: a meta-analysis of direct comparisons. World Psychiatry 12(2):137–148. 10.1002/wps.20038 23737423 10.1002/wps.20038 PMC3683266

[44] Öst L-G, Enebrink P, Finnes A, Ghaderi A, Havnen A, Kvale G et al (2022) Cognitive behavior therapy for obsessive-compulsive disorder in routine clinical care: a systematic review and meta-analysis. Behav Res Ther 159:104170. 10.1016/j.brat.2022.104170 36302283 10.1016/j.brat.2022.104170

[45] Linardon J, Kothe EJ, Fuller-Tyszkiewicz M (2019) Efficacy of psychotherapy for bulimia nervosa and binge‐eating disorder on self‐esteem improvement: Meta‐analysis. Eur Eat Disord Rev 27(2):109–123. 10.1002/erv.2662 30623519 10.1002/erv.2662

[46] Svaldi J, Schmitz F, Baur J, Hartmann AS, Legenbauer T, Thaler C et al (2019) Efficacy of psychotherapies and pharmacotherapies for bulimia nervosa. Psychol Med 49(6):898–910. 10.1017/S0033291718003525 30514412 10.1017/S0033291718003525

[47] Linardon J (2018) Rates of abstinence following psychological or behavioral treatments for binge-eating disorder: Meta‐analysis. Int J Eat Disord 51(8):785–797. 10.1002/eat.22897 30058074 10.1002/eat.22897

[48] Öst LG, Brattmyr M, Finnes A, Ghaderi A, Havnen A, Hedman-Lagerlöf M et al (2024) Cognitive behavior therapy for adult eating disorders in routine clinical care: a systematic review and meta‐analysis. Int J Eat Disord 57(2):249–264. 10.1002/eat.24104 38098336 10.1002/eat.24104

[49] A-tjak JG, Davis ML, Morina N, Powers MB, Smits JA, Emmelkamp PM (2015) A meta-analysis of the efficacy of acceptance and commitment therapy for clinically relevant mental and physical health problems. S Karger AG Basel Switz 30–36. 10.1159/000365764 10.1159/000365764 25547522

[50] Öst L-G, Riise EN, Wergeland GJ, Hansen B, Kvale G (2016) Cognitive behavioral and pharmacological treatments of OCD in children: a systematic review and meta-analysis. J Anx Disord 43:58–69. 10.1016/j.janxdis.2016.08.003 10.1016/j.janxdis.2016.08.003 27632568

[51] Cuijpers P, Karyotaki E, Weitz E, Andersson G, Hollon SD, van Straten A (2014) The effects of psychotherapies for major depression in adults on remission, recovery and improvement: a meta-analysis. J Affect Disord 159:118–126. 10.1016/j.jad.2014.02.026 24679399 10.1016/j.jad.2014.02.026

[52] Page MJ, McKenzie JE, Bossuyt PM, Boutron I, Hoffmann TC, Mulrow CD et al (2021) The PRISMA 2020 statement: an updated guideline for reporting systematic reviews. BMJ 372. 10.1136/bmj.n71 10.1136/bmj.n71 PMC8005924 33782057

[53] Öst L-G (2008) Efficacy of the third wave of behavioral therapies: a systematic review and meta-analysis. Behav Res Ther 46(3):296–321. 10.1016/j.brat.2007.12.005 18258216 10.1016/j.brat.2007.12.005

[54] Cicchetti DV (1994) Guidelines, criteria, and rules of thumb for evaluating normed and standardized assessment instruments in psychology. Psychol Assess 6(4):284–290. 10.1037/1040-3590.6.4.284

[55] Sterne JAC, Savovic J, Page MJ, Elbers RG, Blencowe NS, Boutron I et al (2019) RoB 2: a revised tool for assessing risk of bias in randomised trials. BMJ 366:l4898. 10.1136/bmj.l4898 31462531 10.1136/bmj.l4898

[56] Sterne JA, Hernán MA, Reeves BC, Savović J, Berkman ND, Viswanathan M et al (2016) ROBINS-I: a tool for assessing risk of bias in non-randomised studies of interventions. BMJ 355:i4919. 10.1136/bmj.i4919 27733354 10.1136/bmj.i4919 PMC5062054

[57] Cooper Z, Fairburn C (1987) The eating disorder examination: a semi-structured interview for the assessment of the specific psychopathology of eating disorders. Int J Eat Disord 6(1):1–8. 10.1080/21662630.2013.840119

[58] Fairburn CG, Beglin SJ (1994) Assessment of eating disorders: interview or self-report questionnaire? Int J Eat Disord 16(4):363–370. 10.1002/1098-108X 7866415

[59] Garner D, Olmsted M (1984) Eating disorder inventory (EDI) manual. Psychological Assessment Resources, Florida

[60] Garner DM (2004) EDI-3, eating disorder inventory-3: Professional manual. Psychological Assessment Resources, Incorporated, Odessa, FLA

[61] Kovacs M (1992) Children’s Depression Inventory Manual. Multi-Health Systems. Inc, New York

[62] Beck AT, Ward CH, Mendelson M, Mock J, Erbaugh J (1961) An inventory for measuring depression. Arch Gen Psych 4(6):561–571. 10.1001/archpsyc.1961.01710120031004 10.1001/archpsyc.1961.01710120031004 13688369

[63] Beck AT, Steer RA, BDI (1993) ed Beck depression inventory: manual. San Antonio, Tex

[64] Angold A, Costello EJ, Messer SC, Pickles A (1995) Development of a short questionnaire for use in epidemiological studies of depression in children and adolescents. Int J Methods Psychiatr Res 5(4):237–249

[65] Lakens D (2013) Calculating and reporting effect sizes to facilitate cumulative science: a practical primer for t-tests and ANOVAs. Front Psychol 4:62627. 10.3389/fpsyg.2013.00863 10.3389/fpsyg.2013.00863 PMC3840331 24324449

[66] Lipsey MW, Wilson DB (2001) Practical meta-analysis. Practical meta-analysis. Sage Publications, Inc, Thousand Oaks, CA, US

[67] Borenstein M, Hedges L, Higgins J, Rothstein H (2022) Comprehensive Meta-Analysis, 4 edn. Biostat, Englewood, NJ

[68] Lipsey MW (1990) Design sensitivity: statistical power for experimental research. sage

[69] Sawilowsky SS (2009) New effect size rules of thumb. J Mod Appl Stat Methods 8:597–599. 10.56801/10.56801/v8.i.452

[70] Borenstein M (2022) In a meta-analysis, the I-squared statistic does not tell us how much the effect size varies. J Clin Epidemiol 152:281–284. 10.1016/j.jclinepi.2022.10.003 36223816 10.1016/j.jclinepi.2022.10.003

[71] Egger M, Smith G, Schneider M, Minder C (1998) Bias in meta-analysis detected by a simple, graphical test. BMJ 316(7129):469. 10.1136/bmj.316.7129.469 10.1136/bmj.315.7109.629 PMC2127453 9310563

[72] Duval S, Tweedie R (2000) Trim and fill: a simple funnel-plot-based method of testing and adjusting for publication bias in meta-analysis. Biometrics 56(2):455–463. 10.1111/j.0006-341x.2000.00455.x 10877304 10.1111/j.0006-341x.2000.00455.x

[73] Öst L-G, Enebrink P, Finnes A, Ghaderi A, Havnen A, Kvale G et al (2023) Cognitive behavior therapy for adult depressive disorders in routine clinical care: a systematic review and meta-analysis. J Affect Disord 331:322–333. 10.1016/j.jad.2023.03.002 36894029 10.1016/j.jad.2023.03.002

[74] Öst L-G, Enebrink P, Finnes A, Ghaderi A, Havnen A, Kvale G et al (2023) Cognitive behavior therapy for adult post-traumatic stress disorder in routine clinical care: a systematic review and meta-analysis. Behav Res Ther 166:104323. 10.1016/j.brat.2023.104323 37257304 10.1016/j.brat.2023.104323

[75] Öst L-G, Enebrink P, Finnes A, Ghaderi A, Havnen A, Kvale G et al Cognitive behavior therapy for adult anxiety disorders in routine clinical care: a systematic review and meta-analysis. Clin Psychol Sci Pract. 30(3), 272–290. 10.1037/cps0000144

[76] Jaccard J, Guilamo-Ramos V (2002) Analysis of variance frameworks in clinical child and adolescent psychology: issues and recommendations. J Clin Child Adolesc Psychol 31(1):130–146. 10.1207/S15374424JCCP3101_15 11845645 10.1207/S15374424JCCP3101_15

[77] Valentine JC, Pigott TD, Rothstein HR (2010) How many studies do you need? A primer on statistical power for meta-analysis. J Educ Behav Stat 35(2):215–247. 10.3102/1076998609346961

[78] Lock J (2015) An update on evidence-based psychosocial treatments for eating disorders in children and adolescents. J Clin Child Adolesc Psychol 44(5):707–721. 10.1080/15374416.2014.971458 25580937 10.1080/15374416.2014.971458

[79] Fernández GG, Díaz AK, Udeanu A (2023) Effectiveness of psychological interventions for eating disorders in adolescence: an overview of systematic reviews. Rev Psicol Clin Con Ninos Adolesc 10(1):11. 10.21134/rpcna.2023.10.1.10

[80] Couturier J, Kimber M, Szatmari P (2013) Efficacy of family-based treatment for adolescents with eating disorders: a systematic review and meta‐analysis. Int J Eat Disord 46(1):3–11. 10.1002/eat.22042 22821753 10.1002/eat.22042

[81] Lock J, Couturier J, Bryson S, Agras S (2006) Predictors of dropout and remission in family therapy for adolescent anorexia nervosa in a randomized clinical trial. Int J Eat Disord 39(8):639–647. 10.1002/eat.20328 16927385 10.1002/eat.20328

[82] Russell H, Aouad P, Le A, Marks P, Maloney D, Touyz S, Maguire S (2023) Psychotherapies for eating disorders: findings from a rapid review. J Eat Disord 11(1):175. 10.1186/s40337-023-00886-w 37794513 10.1186/s40337-023-00886-w PMC10548609

[83] Wergeland GJH, Fjermestad KW, Marin CE, Haugland BSM, Silverman WK, Öst LG et al (2015) Predictors of dropout from community clinic child CBT for anxiety disorders. J Anx Disord 31:1–10. 10.1016/j.janxdis.2015.01.004 10.1016/j.janxdis.2015.01.004 25637909

[84] Hamadi L, Holliday J (2020) Moderators and mediators of outcome in treatments for anorexia nervosa and bulimia nervosa in adolescents: a systematic review of randomized controlled trials. Int J Eat Disord 53(1):3–19. 10.1002/eat.23159 31506978 10.1002/eat.23159

[85] Le Grange D, Lock J, Agras WS, Moye A, Bryson SW, Jo B, Kraemer HC (2012) Moderators and mediators of remission in family-based treatment and adolescent focused therapy for anorexia nervosa. Behav Res Ther 50(2):85–92. 10.1016/j.brat.2011.11.003 22172564 10.1016/j.brat.2011.11.003 PMC3260378

[86] Ciao AC, Accurso EC, Fitzsimmons-Craft EE, Le Grange D (2015) Predictors and moderators of psychological changes during the treatment of adolescent bulimia nervosa. Behav Res Ther 69:48–53. 10.1016/j.brat.2015.04.002 25874955 10.1016/j.brat.2015.04.002 PMC4428338

[87] Lock J, Le Grange D (2019) Family-based treatment: where are we and where should we be going to improve recovery in child and adolescent eating disorders. Int J Eat Disord 52(4):481–487. 10.1002/eat.22980 30520532 10.1002/eat.22980

[88] Ball J, Mitchell P (2004) A randomized controlled study of cognitive behavior therapy and behavioral family therapy for anorexia nervosa patients. Eat Disord 12(4):303–314. 10.1080/10640260490521389 16864523 10.1080/10640260490521389

[89] Schmidt U, Lee S, Beecham J, Perkins S, Treasure J, Yi I et al (2007) A randomized controlled trial of family therapy and cognitive behavior therapy guided self-care for adolescents with bulimia nervosa and related disorders. Am J Psychiatry 164(4):591–598. 10.1176/ajp.2007.164.4.591 17403972 10.1176/ajp.2007.164.4.591

[90] Le Grange D, Lock J, Agras WS, Bryson SW, Jo B (2015) Randomized clinical trial of family-based treatment and cognitive-behavioral therapy for adolescent bulimia nervosa. J Am Acad Child Adolesc Psychiatry 54(11):886–894 e2. 10.1016/j.jaac.2015.08.008 26506579 10.1016/j.jaac.2015.08.008 PMC4624104

