# Supplementary material for: Family therapy and cognitive behavior therapy for eating disorders in children and adolescents in routine clinical care: a systematic review and meta-analysis
Source: Eur Child Adolesc Psychiatry. 2024 Aug 27;34(3):883–902. doi: 10.1007/s00787-024-02544-1 (PMC11909078; doi:10.1007/s00787-024-02544-1)
Supplement: Supplementary file 1 — Supplementary Material 1 [file 787_2024_2544_MOESM1_ESM.docx]

Family therapy and cognitive behavior therapy for eating disorders in children and adolescents in routine clinical care: a systematic review and meta-analysis

Gro Janne Wergeland^1,2^, Ata Ghaderi^3^, Krister Fjermestad^4^, Pia Enebrink^3^, Lillan Halsaa^4^, Urdur Njardvik^5^, Eili N. Riise^6^, Gyri Vorren^1^, & Lars-Göran Öst^7^

**Supporting information**

1. PRISMA checklist

2. Full search strategy

3. References to efficacy studies

4. References to included studies

5. Risk of bias classification

**S1: PRISMA checklist**

| **Section/topic** | **#** | **Checklist item** | **Reported on page** |
| --- | --- | --- | --- |
| Title | 1 | Identify the report as a systematic review, meta-analysis, or both. | 1 |
| **ABSTRACT** | | |  |
| Structured summary | 2 | Provide a structured summary including, as applicable: background; objectives; data sources; study eligibility criteria, participants, and interventions; study appraisal and synthesis methods; results; limitations; conclusions and implications of key findings; systematic review registration number. | 3 |
| **INTRODUCTION** | | |  |
| Rationale | 3 | Describe the rationale for the review in the context of what is already known. | 4-7 |
| Objectives | 4 | Provide an explicit statement of questions being addressed with reference to participants, interventions, comparisons, outcomes, and study design (PICOS). | 7-8 |
| **METHODS** | | |  |
| Protocol and registration | 5 | Indicate if a review protocol exists, if and where it can be accessed (e.g., Web address), and, if available, provide registration information including registration number. | 3,7 |
| Eligibility criteria | 6 | Specify study characteristics (e.g., PICOS, length of follow-up) and report characteristics (e.g., years considered, language, publication status) used as criteria for eligibility, giving rationale. | 8 |
| Information sources | 7 | Describe all information sources (e.g., databases with dates of coverage, contact with study authors to identify additional studies) in the search and date last searched. | 8 |
| Search | 8 | Present full electronic search strategy for at least one database, including any limits used, such that it could be repeated. | Supplement S2 |
| Study selection | 9 | State the process for selecting studies (i.e., screening, eligibility, included in systematic review, and, if applicable, included in the meta-analysis). | 8-9  Figure 1  Suppl. S4 |
| Data collection process | 10 | Describe method of data extraction from reports (e.g., piloted forms, independently, in duplicate) and any processes for obtaining and confirming data from investigators. | 10-11 |
| Data items | 11 | List and define all variables for which data were sought (e.g., PICOS, funding sources) and any assumptions and simplifications made. | 10-11 |
| Risk of bias in individual studies | 12 | Describe methods used for assessing risk of bias of individual studies (including specification of whether this was done at the study or outcome level), and how this information is to be used in any data synthesis. | 11-12 |
| Measures | 13 | State the principal summary measures (e.g., risk ratio, difference in means). | 13-15 |
| Synthesis of results | 14 | Describe the methods of handling data and combining results of studies, if done, including measures of consistency (e.g., I^2^) for each meta-analysis. | 13-15 |
| Risk of bias across studies | 15 | Specify any assessment of risk of bias that may affect the cumulative evidence (e.g., publication bias, selective reporting within studies). | 16-17 |
| Additional analyses | 16 | Describe methods of additional analyses (e.g., sensitivity or subgroup analyses, meta-regression), if done, indicating which were pre-specified. | 18-19 |
| **Section/topic** | **#** | **Checklist item** | **Reported on page** |
| **RESULTS** | | |  |
| Study selection | 17 | Give numbers of studies screened, assessed for eligibility, and included in the review, with reasons for exclusions at each stage, ideally with a flow diagram. | Figure 1, |
| Study characteristics | 18 | For each study, present characteristics for which data were extracted (e.g., study size, PICOS, follow-up period) and provide the citations. | 15-16,  Table 1-2 |
| Risk of bias within studies | 19 | Present data on risk of bias of each study and, if available, any outcome level assessment (see item 12). | 16-17,  Suppl. S5 |
| Results of individual studies | 20 | For all outcomes considered (benefits or harms), present, for each study: (a) simple summary data for each intervention group (b) effect estimates and confidence intervals, ideally with a forest plot. | 17-21,  Table 3-8 |
| Synthesis of results | 21 | Present results of each meta-analysis done, including confidence intervals and measures of consistency. | 17-21,  Table 3-8 |
| Risk of bias across studies | 22 | Present results of any assessment of risk of bias across studies (see Item 15). | 17. |
| Additional analysis | 23 | Give results of additional analyses, if done (e.g., sensitivity or subgroup analyses, meta-regression [see Item 16]). | 18-19 |
| **DISCUSSION** | | |  |
| Summary of evidence | 24 | Summarize the main findings including the strength of evidence for each main outcome; consider their relevance to key groups (e.g., healthcare providers, users, and policy makers). | 21-16 |
| Limitations | 25 | Discuss limitations at study and outcome level (e.g., risk of bias), and at review-level (e.g., incomplete retrieval of identified research, reporting bias). | 25-26 |
| Conclusions | 26 | Provide a general interpretation of the results in the context of other evidence, and implications for future research. | 26 |
| **FUNDING** | | |  |
| Funding | 27 | Describe sources of funding for the systematic review and other support; role of funders for the systematic review. | Enclosed document |

**S2. Full search strategy**

## Ovid MEDLINE(R) and Epub Ahead of Print, In-Process, In-Data-Review & Other Non-Indexed Citations and Daily <1946 to December 07, 2023>; Search date 8 Dec 2023

1 cognitive behavioral therapy/ 30726

2 (CBT or CBT-ED or "cognitive behavio?r* therap*" or "behavio?r* therap*" or "cognitive therap*" or "cognitive behavio?r* treatment*").ti,ab,kf. 39885

3 family therapy/ 9208

4 ((Family* or conjoint or multimodal) adj2 (therap* or Counseling or intervention* or psychotherapy or treatment*)).ti,ab,kf. 26588

5 FT-AN.ti,ab,kf. 33

6 1 or 2 or 3 or 4 or 5 82792

7 "feeding and eating disorders"/ or anorexia nervosa/ or avoidant restrictive food intake disorder/ or binge-eating disorder/ or bulimia nervosa/ or diabulimia/ or "feeding and eating disorders of childhood"/ or food addiction/ or night eating syndrome/ or orthorexia nervosa/ 35331

8 (feeding disorder* or eating disorder* or anorexi* or (avoidant adj2 restrictive food intake disorder*) or ARFID or binge-eating disorder* or binge eating disorder* or bulimi* or diabulimia or food addiction or night eating syndrome or orthorexia or emotional eating or food aversion or food refusal or purging disorder* or "rumination eating" or OSFED or appetite disorder*).ti,ab,kf. 63972

9 7 or 8 71582

10 6 and 9 3371

11 limit 10 to "therapy (maximizes sensitivity)" 1305

12 feasibility studies/ or pilot projects/ or exp clinical study/ 1326545

13 (RCT or random* or non-random* or NRS or pre post study or open trial or open study or clinical study or intervention study or community trial).ti,ab,kf. 1557518

14 12 or 13 2344117

15 10 and 14 1165

16 11 or 15 1419

17 16 not (exp Adult/ not (adolescent/ or child/)) 965

Comments and explanations:
/ = subject heading
.ti,ab,kw. = search words either in title or abstract or authors’ keywords

Line 11 = filter for finding therapy studies in Medline Ovid, from Nancy L. Wilczynski PhD and R. Brian Haynes MD, PhD et al. of the Health Information Research Unit (HIRU) at McMaster University. The search words are: clinical trial.mp. OR clinical trial.pt. OR random:.mp. OR tu.xs.

(.mp. = multi purpose = several fields are searched; .pt. = publication type; random: = random* ; tu.xs. = therapeutic use as subheading)
from <http://hiru.mcmaster.ca/hiru/HIRU_Hedges_home.aspx>

Link: <https://ovidsp.ovid.com/ovidweb.cgi?T=JS&NEWS=N&PAGE=main&SHAREDSEARCHID=3bS84lfAEfEMYvwNsUEKRaJs0uMu5UtOBx1gEDgHxulCeoNd7atXXPGfQ7kPUeBTI>

## Embase (OVID) <1974 to 2023 December 07>; Search date 8 Dec 2023

1 cognitive behavioral therapy/ 25719

2 cognitive therapy/ 44637

3 behavior therapy/ or exposure therapy/ or "desensitization (psychology)"/ or implosive therapy/ or systematic desensitization/ or virtual reality exposure therapy/ 49425

4 (CBT or CBT-ED or "cognitive behavio?r* therap*" or "behavio?r* therap*" or "cognitive therap*" or "cognitive behavio?r* treatment*").ti,ab,kf. 58373

5 family therapy/ 14240

6 ((Family* or conjoint or multimodal) adj2 (therap* or Counseling or intervention* or psychotherapy or treatment*)).ti,ab,kf. 38002

7 FT-AN.ti,ab,kf. 58

8 1 or 2 or 3 or 4 or 5 or 6 or 7 160693

9 eating disorder/ or anorexia nervosa/ or avoidant restrictive food intake disorder/ or binge eating disorder/ or bulimia/ or emotional eating/ or food addiction/ or food aversion/ or food refusal/ or orthorexia/ or purging disorder/ 63164

10 (feeding disorder* or eating disorder* or anorexi* or (avoidant adj2 restrictive food intake disorder*) or ARFID or binge-eating disorder* or binge eating disorder* or bulimi* or diabulimia or food addiction or night eating syndrome or orthorexia or emotional eating or food aversion or food refusal or purging disorder* or "rumination eating" or OSFED or appetite disorder*).ti,ab,kf. 88050

11 9 or 10 104872

12 8 and 11 6840

13 limit 12 to "therapy (maximizes sensitivity)" 3373

14 clinical study/ or community trial/ or intervention study/ or open study/ 282174

15 (RCT or random* or non-random* or NRS or pre post study or open trial or open study or clinical study or intervention study or community trial).ti,ab,kf. 2129960

16 14 or 15 2336189

17 12 and 16 1389

18 13 or 17 3429

19 18 not (exp adult/ not (exp child/ or exp adolescent/)) 2261

20 limit 19 to (books or chapter or conference abstract) 104

21 19 not 20 2157

Comment on line 13: Filter for RCT by Haynes:
random:.tw. OR clinical trial:.mp. OR exp health care quality/
[**http://hiru.mcmaster.ca/hiru/HIRU_Hedges_home.aspx**](http://hiru.mcmaster.ca/hiru/HIRU_Hedges_home.aspx)

Link:

<https://ovidsp.ovid.com/ovidweb.cgi?T=JS&NEWS=N&PAGE=main&SHAREDSEARCHID=458qLVpwdmXsabpLXgIkBbVzfcIekNx4B0S529jEautkXgGTqqiWs7IYRjHwmWLl>

APA PsycInfo (OVID) <1806 to November Week 4 2023> Search date 8 Dec 2023

1 cognitive behavior therapy/ 24839

2 behavior therapy/ or exp aversion therapy/ or conversion therapy/ or dialectical behavior therapy/ or exp exposure therapy/ or implosive therapy/ or reciprocal inhibition therapy/ or "response cost"/ or systematic desensitization therapy/ 24221

3 cognitive therapy/ 14107

4 (CBT or CBT-ED or "cognitive behavio?r* therap*" or "behavio?r* therap*" or "cognitive therap*" or "cognitive behavio?r* treatment*").tw. 54618

5 family therapy/ or conjoint therapy/ or strategic family therapy/ or structural family therapy/ 23554

6 ((Family* or conjoint or multimodal) adj2 (therap* or Counseling or intervention* or psychotherapy or treatment*)).tw. 37518

7 FT-AN.tw. 14

8 1 or 2 or 3 or 4 or 5 or 6 or 7 114246

9 eating disorders/ or anorexia nervosa/ or binge eating disorder/ or bulimia/ or feeding disorders/ or "purging (eating disorders)"/ or "rumination (eating)"/ 35372

10 (feeding disorder* or eating disorder* or anorexi* or (avoidant adj2 restrictive food intake disorder*) or ARFID or binge-eating disorder* or binge eating disorder* or bulimi* or diabulimia or food addiction or night eating syndrome or orthorexia or emotional eating or food aversion or food refusal or purging disorder* or "rumination eating" or OSFED or appetite disorder*).tw. 46713

11 9 or 10 48696

12 8 and 11 5084

13 limit 12 to "therapy (maximizes sensitivity)" 4778

14 (RCT or random* or non-random* or NRS or pre post study or open trial or open study or clinical study or intervention study or community trial).tw. 255456

15 12 and 14 859

16 13 or 15 4779

17 limit 16 to "300 adulthood <age 18 yrs and older>" 2027

18 limit 17 to (100 childhood <birth to age 12 yrs> or 200 adolescence <age 13 to 17 yrs>) 648

19 16 not (17 not 18) 3400

20 limit 19 to ("0200 book" or "0240 authored book" or "0280 edited book" or "0300 encyclopedia" or "0400 dissertation abstract") 1235

21 19 not 20 2165

Comments:

Line 13: Filter for effect studies: control:.tw. OR random:.tw. OR exp treatment
see: <https://hiru.mcmaster.ca/hiru/HIRU_Hedges_PsycINFO_Strategies.aspx>

The Textword (TW) field is an alias for all of the fields in the database that contain text and are appropriate for a free-text subject search. The Text word fields in APA PsycInfo® include [Table of Contents](https://ospguides.ovid.com/OSPguides/psycdb.htm?S=JJPFFPGHMNACNJIDKPMJMGMIONLGAA00#TC) (TC), [Title](https://ospguides.ovid.com/OSPguides/psycdb.htm?S=JJPFFPGHMNACNJIDKPMJMGMIONLGAA00#TI) (TI), [Abstract](https://ospguides.ovid.com/OSPguides/psycdb.htm?S=JJPFFPGHMNACNJIDKPMJMGMIONLGAA00#AB) (AB), and [Key Concepts](https://ospguides.ovid.com/OSPguides/psycdb.htm?S=JJPFFPGHMNACNJIDKPMJMGMIONLGAA00#ID) (ID).

Link:

<https://ovidsp.ovid.com/ovidweb.cgi?T=JS&NEWS=N&PAGE=main&SHAREDSEARCHID=6c5xO330jVlYuxqP1zVgLTfsh41pHPEd8re4pcCaiQMzxS4RLPwXF4kvtaMDYF1QX>

**S3. References to efficacy studies**

Agras, W., Lock, J., Brandt, H., Bryson, S., Dodge, E., Halmi, K., . . . Woodside, B. (2014). Comparison of 2 family therapies for adolescent anorexia nervosa: A randomized parallel trial. *JAMA Psychiatry, 72*, 1279–1286.

Ball, J., & Mitchell, P. (2004) A randomized controlled study of cognitive behavior therapy and behavioral family therapy for anorexia nervosa patients, *Eating Disorders, 12*, 303-314, DOI: 10.1080/10640260490521389

Gowers, S., Clark, A., Roberts, C., Griffiths, A., Edwards, V., Bryan, C., . . . Barrett, B. (2007). Clinical effectiveness of treatments for anorexia nervosa in adolescents: Randomised controlled trial. *The British Journal of Psychiatry, 191*, 427–435. doi:10.1192=bjp.bp. 107.036764

Hilbert, A., Petroff, D., Neuhaus, P., & Schmidt, R. (2020). Cognitive-behavioral therapy for adolescents with an age-adapted diagnosis of binge-eating disorder: A randomized clinical trial. *Psychotherapy and Psychosomatics*, *89*, 51–53. <https://doi.org/10.1159/> 00503116

Jones, M., Luce, K. H., Osborne, M. I., Taylor, K., Cunning, D., Doyle, A. C., . . . Taylor, C. B. (2008). Randomized, controlled trial of an internet-facilitated intervention for reducing binge eating and overweight in adolescents. *Pediatrics, 121*, 453–462. doi:10.1542=peds.2007-1173

Le Grange, D., Crosby, R. D., Rathouz, P. J., & Leventhal, B. L. (2007). A randomized controlled comparison of family-based treatment and supportive psychotherapy for adolescent bulimia nervosa. *Archives of General Psychiatry, 64*, 1049–1056.

Le Grange, D., Lock, J., Agras, W. S., Bryson, S. W., & Jo, B. (2015). Randomized clinical trial of family-based treatment and cognitive-behavioral therapy for adolescent bulimia nervosa. *Journal of the American Academy of Child and Adolescent Psychiatry*, *54*, 886–894. <https://doi.org/10.1016/j.jaac.2015.08.008>

Lock, J., Agras, W. S., Bryson, S., & Kraemer, H. C. (2005). A comparison of short- and long-term family therapy for adolescent anorexia nervosa. *Journal of the American Academy of Child & Adolescent Psychiatry, 44*, 632–639.

Lock, J., Couturier, J., Matheson, B. E., Datta, N., Citron, K., Sami, S., Welch, H., Webb, C., Doxtdator, K., & John-Carson, N. (2021). Feasibility of conducting a randomized controlled trial comparing family-based treatment via videoconferencing and online guided self-help family-based treatment for adolescent anorexia nervosa. *International Journal of Eating Disorders*, *54*, 1998–2008. <https://doi.org/10.1002/eat.23611>

Lock, J., Le Grange, D., Agras, W. S., Fitzpatrick, K. K., Jo, B., Accurso, E., Forsberg, S., Anderson, K., Arnow, K., & Stainer, M. (2015). Can adaptive treatment improve outcomes in family-based therapy for adolescents with anorexia nervosa? Feasibility and treatment effects of a multi-site treatment study. *Behaviour Research and Therapy*, *44*, 90–95. <https://doi.org/10.1016/j.brat.2015.07.015>

Lock, J., Le Grange, D., Agras, W. S., Moye, A., Bryson, S. W., & Jo, B. (2010). Randomized clinical trial comparing family-based treatment with adolescent-focused individual therapy for adolescents with anorexia nervosa. *Archives of General Psychiatry, 67*, 1025–1032. doi:10.1001=archgenpsychiatry.2010.128

Lock, J., Sadeh-Sharvit, S., & L’Insalata, A. (2019). Feasibility of conducting a randomized clinical trial using family-based treatment for avoidant/restrictive food intake disorder. *International Journal of Eating Disorders*, *52*, 746–751. <https://doi.org/10.1002/eat>. 23077

Robin, A. L., Siegel, P. T., Moye, A. W., Gilroy, M., Dennis, A. B., & Sikand, A. (1999). A controlled comparison of family versus individual therapy for adolescents with anorexia nervosa. *Journal of the American Academy of Child and Adolescent Psychiatry,* *38*, 1482–1489.

Russell, G. F. M., Szmukler, G. I., Dare, C., & Eisler, I. (1987). An evaluation of family therapy in anorexia nervosa and bulimia nervosa. *Archives of General Psychiatry, 44*, 1047–1056.

Stefini, A., Salzer, S., Reich, G., Horn, H., Winkelmann, K., Bents, H., Rutz, U., Frost, U., von Boetticher, A., Ruhl, U., Specht, N., & Kronmuller, K. T. (2017). Cognitive behavioral and psychodynamic therapy in female adolescents with bulimia nervosa: A randomized controlled trial. *Journal of the American Academy of Child and Adolescent Psychiatry*, *56*, 329–335. <https://doi.org/10.1016/j.jaac>. 2017.01.019

**S4. References to included studies**

Accurso, E. C., Astrachan-Fletcher, E., O’Brien, S., McClanahan, S. F., & Le Grange, D. (2018). Adaptation and implementation of family-based treatment enhanced with dialectical behavior therapy skills for anorexia nervosa in community-based specialist clinics. *Eating disorders*, *26*(2), 149-163.

Anastasiadou, D., Folkvord, F., Brugnera, A., Canas Vinader, L., SerranoTroncoso, E., Carretero Jardi, C., ... & Lupiañez‐Villanueva, F. (2020). An mHealth intervention for the treatment of patients with an eating disorder: a multicenter randomized controlled trial. *International Journal of Eating Disorders*, *53*(7), 1120-1131.

Anderson, K. E., Byrne, C. E., Crosby, R. D., & Le Grange, D. (2017). Utilizing telehealth to deliver family‐based treatment for adolescent anorexia nervosa. *International Journal of Eating Disorders*, *50*(10), 1235-1238.

Bentz, M., Pedersen, S. H., & Moslet, U. (2021). An evaluation of family-based treatment for restrictive-type eating disorders, delivered as standard care in a public mental health service. *Journal of Eating Disorders*, *9*, 1-12.

Calugi, S., & Dalle Grave, R. (2019). Body image concern and treatment outcomes in adolescents with anorexia nervosa. *International Journal of Eating Disorders*, *52*(5), 582-585.

Chew, C. S. E., Kelly, S., Tay, E. E., Baeg, A., Khaider, K. B., Oh, J. Y., ... & Davis, C. (2021). Implementation of family‐based treatment for Asian adolescents with anorexia nervosa: A consecutive cohort examination of outcomes. *International Journal of Eating Disorders*, *54*(1), 107-116.

Coelho, J. S., Beach, B., O'Brien, K., Marshall, S., & Lam, P. Y. (2019). Effectiveness of family-based treatment for pediatric eating disorders in a tertiary care setting. *Clinical Practice in Pediatric Psychology*, *7*(2), 105

Couturier, J., Isserlin, L., & Lock, J. (2010). Family-based treatment for adolescents with anorexia nervosa: A dissemination study. *Eating Disorders*, *18*(3), 199-209.

Craig, M., Waine, J., Wilson, S., & Waller, G. (2019). Optimizing treatment outcomes in adolescents with eating disorders: the potential role of cognitive behavioral therapy. *International journal of eating disorders*, *52*(5), 538-542.

Dalle Grave, R., Calugi, S., El Ghoch, M., Conti, M., & Fairburn, C. G. (2014). Inpatient cognitive behavior therapy for adolescents with anorexia nervosa: immediate and longer-term effects. *Frontiers in psychiatry*, *5*, 71925.

Dalle Grave, R., Calugi, S., Sartirana, M., & Fairburn, C. G. (2015). Transdiagnostic cognitive behaviour therapy for adolescents with an eating disorder who are not underweight. *Behaviour research and therapy*, *73*, 79-82.

Dalle Grave, R., Sartirana, M., & Calugi, S. (2019). Enhanced cognitive behavioral therapy for adolescents with anorexia nervosa: Outcomes and predictors of change in a real‐world setting. *International Journal of Eating Disorders*, *52*(9), 1042-1046.

Dalle Grave, R., Conti, M., & Calugi, S. (2020). Effectiveness of intensive cognitive behavioral therapy in adolescents and adults with anorexia nervosa. *International Journal of Eating Disorders*, *53*(9), 1428-1438.

Dalle Grave, R., Sartirana, M., Dalle Grave, A., & Calugi, S. (2023). Effectiveness of enhanced cognitive behaviour therapy for patients aged 14 to 25: A promising treatment for anorexia nervosa in transition‐age youth. *European Eating Disorders Review*.

Eisler, I., Simic, M., Hodsoll, J., Asen, E., Berelowitz, M., Connan, F., ... & Landau, S. (2016). A pragmatic randomised multi-centre trial of multifamily and single family therapy for adolescent anorexia nervosa. *BMC psychiatry*, *16*, 1-14.

Gabel, K., Pinhas, L., Eisler, I., Katzman, D., & Heinmaa, M. (2014). The effect of multiple family therapy on weight gain in adolescents with anorexia nervosa: Pilot data. *Journal of the Canadian academy of child and adolescent psychiatry*, *23*(3), 196.

Geist, R., Heinmaa, M., Stephens, D., Davis, R., & Katzman, D. K. (2000). Comparison of family therapy and family group psychoeducation in adolescents with anorexia nervosa. *The Canadian Journal of Psychiatry*, *45*(2), 173-178.

Gelin, Z., Fuso, S., Hendrick, S., Cook‐Darzens, S., & Simon, Y. (2015). The effects of a multiple family therapy on adolescents with eating disorders: an outcome study. *Family process*, *54*(1), 160-172.

Girz, L., Lafrance Robinson, A., Foroughe, M., Jasper, K., & Boachie, A. (2013). Adapting family‐based therapy to a day hospital programme for adolescents with eating disorders: Preliminary outcomes and trajectories of change. *Journal of Family Therapy*, *35*, 102-120.

Goldstein, M., Peters, L., Baillie, A., McVeagh, P., Minshall, G., & Fitzjames, D. (2011). The effectiveness of a day program for the treatment of adolescent anorexia nervosa. *International Journal of Eating Disorders*, *44*(1), 29-38.

Goldstein, M., Murray, S. B., Griffiths, S., Rayner, K., Podkowka, J., Bateman, J. E., ... & Thornton, C. E. (2016). The effectiveness of family‐based treatment for full and partial adolescent anorexia nervosa in an independent private practice setting: Clinical outcomes. *International Journal of Eating Disorders*, *49*(11), 1023-1026.

Gowers, S. G., Clark, A. F., Roberts, C., Byford, S., Barrett, B., Griffiths, A., ... & Roots, P. (2010). A randomised controlled multicentre trial of treatments for adolescent anorexia nervosa including assessment of cost-effectiveness and patient acceptability-the TOuCAN trial. *Health Technol Assess*, *14*(15), 1-98.

Henderson, K., Buchholz, A., Obeid, N., Mossiere, A., Maras, D., Norris, M., ... & Spettigue, W. (2014). A family-based eating disorder day treatment program for youth: examining the clinical and statistical significance of short-term treatment outcomes. *Eating Disorders*, *22*(1), 1-18.

Hiney-Saunders, K., Ousley, L., Caw, J., Cassinelli, E., & Waller, G. (2021). Effectiveness of treatment for adolescents and adults with anorexia nervosa in a routine residential setting. *Eating Disorders*, *29*(1), 103-117.

Hollesen, A., Clausen, L., & Rokkedal, K. (2013). Multiple family therapy for adolescents with anorexia nervosa: a pilot study of eating disorder symptoms and interpersonal functioning. *Journal of Family Therapy*, *35*, 53-67.

Hughes, E. K., Le Grange, D., Court, A., Yeo, M., Campbell, S., Whitelaw, M., ... & Sawyer, S. M. (2014). Implementation of family-based treatment for adolescents with anorexia nervosa. *Journal of Pediatric Health Care*, *28*(4), 322-330.

Hughes, E. K., Le Grange, D., Court, A., & Sawyer, S. M. (2017). A case series of family‐based treatment for adolescents with atypical anorexia nervosa. *International Journal of Eating Disorders*, *50*(4), 424-432.

Hurst, K., & Zimmer‐Gembeck, M. (2019). Family‐based treatment with cognitive behavioural therapy for anorexia. *Clinical Psychologist*, *23*(1), 61-70.

Le Grange, D., Hughes, E. K., Court, A., Yeo, M., Crosby, R. D., & Sawyer, S. M. (2016). Randomized clinical trial of parent-focused treatment and family-based treatment for adolescent anorexia nervosa. *Journal of the American Academy of Child & Adolescent Psychiatry*, *55*(8), 683-692.

Le Grange, D., Pradel, M., Pogos, D., Yeo, M., Hughes, E. K., Tompson, A., ... & Sawyer, S. M. (2021). Family‐based treatment for adolescent anorexia nervosa: Outcomes of a stepped‐care model. *International Journal of Eating Disorders*, *54*(11), 1989-1997.

Le Grange, D., Eckhardt, S., Dalle Grave, R., Crosby, R. D., Peterson, C. B., Keery, H., ... & Martell, C. (2022). Enhanced cognitive-behavior therapy and family-based treatment for adolescents with an eating disorder: a non-randomized effectiveness trial. *Psychological medicine*, *52*(13), 2520-2530.

Lebow, J., O’Brien, J. R. G., Mattke, A., Narr, C., Geske, J., Billings, M., ... & Sim, L. (2021). A primary care modification of family-based treatment for adolescent restrictive eating disorders. *Eating disorders*, *29*(4), 376-389.

Lebow, J., Mattke, A., Narr, C., Partain, P., Breland, R., Gewirtz O’Brien, J. R., ... & Sim, L. (2021). Can adolescents with eating disorders be treated in primary care? A retrospective clinical cohort study. *Journal of eating disorders*, *9*(1), 55.

Lim, J., White, J., Withington, T., Catania, S., Wilson, D., Knight, P., ... & Krishnamoorthy, G. (2023). Family-based treatment takes longer for adolescents with mental health comorbidities: findings from a community mental health service. *Eating disorders*, *31*(6), 588-609.

Madden, S., Miskovic-Wheatley, J., Wallis, A., Kohn, M., Lock, J., Le Grange, D., ... & Touyz, S. (2015). A randomized controlled trial of in-patient treatment for anorexia nervosa in medically unstable adolescents. *Psychological medicine*, *45*(2), 415-427.

Rosling, A., Salonen Ros, H., & Swenne, I. (2016). One-year outcome and incidence of anorexia nervosa and restrictive eating disorders among adolescent girls treated as out-patients in a family-based setting. *Upsala Journal of Medical Sciences*, *121*(1), 50-59.

Schlegl, S., Diedrich, A., Neumayr, C., Fumi, M., Naab, S., & Voderholzer, U. (2016). Inpatient treatment for adolescents with anorexia nervosa: clinical significance and predictors of treatment outcome. *European Eating Disorders Review*, *24*(3), 214-222.

Schmidt, U., Lee, S., Beecham, J., Perkins, S., Treasure, J., Yi, I., ... & Eisler, I. (2007). A randomized controlled trial of family therapy and cognitive behavior therapy guided self-care for adolescents with bulimia nervosa and related disorders. *American Journal of Psychiatry*, *164*(4), 591-598.

Simic, M., Stewart, C. S., Konstantellou, A., Hodsoll, J., Eisler, I., & Baudinet, J. (2022). From efficacy to effectiveness: child and adolescent eating disorder treatments in the real world (part 1)—treatment course and outcomes. *Journal of eating disorders*, *10*(1), 27.

Spettigue, W., & Norris, M. L. (2019). Feasibility of implementing a family-based inpatient program for adolescents with anorexia nervosa: a retrospective cohort study. *Frontiers in Psychiatry*, *10*, 497694.

Terache, J., Wollast, R., Simon, Y., Marot, M., Van der Linden, N., Franzen, A., & Klein, O. (2023). Promising effect of multi-family therapy on BMI, eating disorders and perceived family functioning in adolescent anorexia nervosa: an uncontrolled longitudinal study. *Eating disorders*, *31*(1), 64-84.

Thompson, H., Hurst, K., Green, H., Watkins, J., Collings, N., & Read, S. (2020). Implementing family based treatment in a child and youth eating disorder program: impact on admissions. *International Journal of Adolescent Medicine and Health*, *32*(6), 20170139.

Van Huysse, J. L., Lock, J., Le Grange, D., & Rienecke, R. D. (2022). Weight gain and parental self-efficacy in a family-based partial hospitalization program. *Journal of eating disorders*, *10*(1), 116.

Zanna, V., Castiglioni, M. C., Criscuolo, M., Chianello, I., Elisei, M., Cinelli, G., ... & Vicari, S. (2017). Day-hospital multifocal﻿ integrated treatment for anorexia nervosa in adolescents: a one-year follow-up. *Journal of child and family studies*, *26*(5), 1460-1471.

**S5. Risk of bias classification**

**RCTs**

| **Study** | **Randomization** | **Missing data** | **Measurement of outcome** | **Selection of the results** |
| --- | --- | --- | --- | --- |
| Anastasiadou, 2020 RCT | L | S | S | L |
| Eisler, 2016 RCT | L | L | L | L |
| Eisler, 2016 RCT | L | L | L | L |
| Geist, 2000 RCT | L | L | L | S |
| Geist, 2000 RCT | L | L | L | S |
| Gowers, 2010 RCT | L | L | L | L |
| Le Grange, 2016 RCT | L | L | S | L |
| Le Grange, 2016 RCT | L | L | S | L |
| Madden, 2015 RCT | L | S | L | L |
| Madden, 2015 RCT | L | S | L | L |
| Schmidt, 2007 RCT | L | L | L | S |
| Schmidt, 2007 RCT | L | L | L | S |

*Note*: L = low RoB, S = some concerns, H = high RoB.

**NSRI/Pre-post trials**

| **Study** | **Confounding** | **Selection bias** | **Classification of interventions** | **Deviations from intended interventions** | **Missing data** | **Measurements of outcomes** | **Selection of the Results** |
| --- | --- | --- | --- | --- | --- | --- | --- |
| Accurso, 2018 | S | S | M | L | S | M | M |
| Anderson, 2017 | S | L | L | M | L | S | M |
| Bentz, 2021 | S | L | M | M | S | S | M |
| Calugi, 2019 | S | S | M | L | M | L | M |
| Chew, 2021 | S | M | M | L | L | M | M |
| Coelho, 2019 | S | C | S | M | S | M | M |
| Couturier, 2010 | S | S | L | L | L | M | M |
| Craig, 2019 | S | L | L | M | L | M | M |
| Dalle Grave, 2014 | S | M | L | L | L | M | M |
| Dalle Grave, 2015 | S | M | L | L | L | M | M |
| Dalle Grave, 2019 | S | L | L | L | L | M | M |
| Dalle Grave, 2020 | S | L | L | L | L | M | M |
| Dalle Grave, 2023 | S | M | L | L | M | M | M |
| Gabel, 2014 | S | C | M | M | S | S | M |
| Gelin, 2015 | S | M | M | M | M | M | M |
| Girz, 2013 | S | M | M | M | L | M | M |
| Goldstein, 2011 | S | M | M | M | M | S | M |
| Goldstein, 2016 | S | S | M | M | L | S | M |
| Henderson, 2014 | S | S | M | M | S | M | M |
| Hiney-Saunders, 2021 | S | M | M | M | L | M | M |
| Hollesen, 2013 | S | S | M | M | S | M | M |
| Hughes, 2014 | S | M | M | M | S | M | M |
| Hughes, 2017 | S | L | L | M | M | M | M |
| Hurst, 2019 | S | M | L | M | L | M | M |
| Le Grange 2021 | S | L | L | M | M | M | M |
| Le Grange 2022 | M | L | L | L | M | M | M |
| Le Grange 2022 | M | L | L | L | M | M | M |
| Lebow 2021 | S | M | M | M | M | S | M |
| Lebow, 2021 | S | S | M | L | M | S | M |
| Lim, 2023 | S | C | M | M | S | S | M |
| Rosling 2013 | S | S | M | M | S | M | M |
| Rosling 2013 | S | S | M | M | S | M | M |
| Schlegl, 2015 | M | S | M | L | L | M | M |
| Simic, 2022 | M | L | M | L | L | M | M |
| Simic, 2022 | M | L | M | L | L | M | M |
| Spettigue, 2019 | M | S | M | M | C | M | M |
| Spettigue, 2019 | M | S | M | M | C | M | M |
| Terache, 2023 | S | M | M | M | S | M | M |
| Thompson, 2020 | S | C | M | M | C | M | M |
| Van Huysse, 2022 | S | S | M | M | S | M | M |
| Zanna, 2017 | S | S | M | M | C | M | M |

Note: L = low, M = moderate, S = serious, C = critical RoB.
